# A Novel Nonantibiotic, *lgt*-Based Selection System for Stable Maintenance of Expression Vectors in Escherichia coli and Vibrio cholerae

**DOI:** 10.1128/AEM.02143-17

**Published:** 2018-01-31

**Authors:** Manuela Terrinoni, Stefan L. Nordqvist, Susanne Källgård, Jan Holmgren, Michael Lebens

**Affiliations:** a Department of Microbiology and Immunology, Institute of Biomedicine, Sahlgrenska Academy at University of Gothenburg, Gothenburg, Sweden; b University of Gothenburg Vaccine Research Institute (GUVAX), Institute of Biomedicine, Sahlgrenska Academy at University of Gothenburg, Gothenburg, Sweden; University of Tartu

**Keywords:** plasmid maintenance, Gram-negative bacteria, essential genes, complementation

## Abstract

Antibiotic selection for the maintenance of expression plasmids is discouraged in the production of recombinant proteins for pharmaceutical or other human uses due to the risks of antibiotic residue contamination of the final products and the release of DNA encoding antibiotic resistance into the environment. We describe the construction of expression plasmids that are instead maintained by complementation of the *lgt* gene encoding a (pro)lipoprotein glyceryl transferase essential for the biosynthesis of bacterial lipoprotein. Mutations in *lgt* are lethal in Escherichia coli and other Gram-negative organisms. The *lgt* gene was deleted from E. coli and complemented by the Vibrio cholerae-derived gene provided in *trans* on a temperature-sensitive plasmid, allowing cells to grow at 30°C but not at 37°C. A temperature-insensitive expression vector carrying the V. cholerae-derived *lgt* gene was constructed, whereby transformants were selected by growth at 39°C. The vector was successfully used to express two recombinant proteins, one soluble and one forming insoluble inclusion bodies. Reciprocal construction was done by deleting the *lgt* gene from V. cholerae and complementing the lesion with the corresponding gene from E. coli. The resulting strain was used to produce the secreted recombinant cholera toxin B subunit (CTB) protein, a component of licensed as well as newly developed oral cholera vaccines. Overall, the *lgt* system described here confers extreme stability on expression plasmids, and this strategy can be easily transferred to other Gram-negative species using the E. coli-derived *lgt* gene for complementation.

**IMPORTANCE** Many recombinant proteins are produced in bacteria from genes carried on autonomously replicating DNA elements called plasmids. These plasmids are usually inherently unstable and rapidly lost. This can be prevented by using genes encoding antibiotic resistance. Plasmids are thus maintained by allowing only plasmid-containing cells to survive when the bacteria are grown in medium supplemented with antibiotics. In the described antibiotic-free system for the production of recombinant proteins, an essential gene is deleted from the bacterial chromosome and instead provided on a plasmid. The loss of the plasmid becomes lethal for the bacteria. Such plasmids can be used for the expression of recombinant proteins. This broadly applicable system removes the need for antibiotics in recombinant protein production, thereby contributing to reducing the spread of genes encoding antibiotic resistance, reducing the release of antibiotics into the environment, and freeing the final products (often used in pharmaceuticals) from contamination with potentially harmful antibiotic residues.

## INTRODUCTION

The most commonly used tools for the selection and maintenance of recombinant plasmids in hosts such as Escherichia coli are antibiotic resistance genes. In the development of systems for the industrial production of recombinant proteins that are usually encoded on expression plasmids, the use of antibiotics is undesirable for several reasons. From the point of view of production efficiency, the expression of antibiotic resistance genes imposes an unnecessary metabolic burden on the cells, resulting in reduced growth rates and lower cell densities (1, 2).

Furthermore, the addition of antibiotics to large-scale cultures is expensive and presents risks of allergy development in the exposed staff. There are also risks that, at worst, the final products may be contaminated with antibiotic residues and, at best, purification and quality control costs increase. Finally, there are the dual dangers of contamination of the environment with antibiotic residues and the horizontal gene transfer of antibiotic resistance genes if the DNA is released into the environment, both of which can contribute to the accelerated emergence of potential antibiotic-resistant pathogens (3–6).

Another voiced concern has been the potential for antibiotic resistance genes to be integrated into the human genome when incorporated into DNA vaccines (7, 8). Due to these considerations, a number of alternative strategies for the maintenance of plasmids without the need for antibiotics have been devised. Various auxotrophic complementation systems, in which a gene involved in the synthesis of an essential metabolite is disrupted by mutation or deletion and complemented by the corresponding gene carried on the plasmid, have been developed and employed in a number of different bacteria. The genes targeted for auxotrophic complementation are in most cases involved in either DNA precursor, amino acid, or cell wall biosynthesis, and mutations are conditionally lethal and usually require complementation in modified growth media to function (1, 9, 10).

Methods based on postsegregational killing exploit naturally occurring plasmid maintenance systems. The expression of a stable toxin from a gene inserted into the chromosome is balanced by the expression of a less stable antitoxin inserted into the plasmid. The loss of the plasmid results in the loss of the antitoxin, and the remaining toxin is lethal to the cells. This system, however, has proven to be ineffective in prolonged culture (11).

Operator repressor titration instead utilizes high-copy-number plasmids carrying a short, nonexpressed *lac* operator sequence that functions as the vector-borne selection marker. The sequence binds the LacI repressor and derepresses a modified essential chromosomal gene. The loss of these types of plasmids leads to the death of the bacterium since the repressor gene is no longer titrated out by the plasmid-borne operator sequences (12).

A final approach is to delete an essential chromosomal gene and to use the expression vector to complement it. This has been used in E. coli with the *infA* gene, which encodes a small protein that is essential for DNA synthesis (13). This approach has two advantages. The first advantage is that it confers extremely high stability to the plasmids since the cells cannot survive without them, and the second is that the cells can be cultured in any growth medium in which the parental strain can grow and do not require any special additives. However, due to the need for the deleted gene for cell survival, a convenient means of maintaining the parental strain and transforming the strain with expression vectors can be problematic.

Similar to the latter approach, we have devised a method in which a gene that is essential under all growth conditions was deleted from the chromosomes of both E. coli and Vibrio cholerae and in each case complemented by a nonhomologous gene with the same function. Since plasmid loss results in cell death regardless of the growth conditions and complementation restores normal growth, this system is highly versatile and requires no additional reagents to function. Furthermore, we have devised a method for the maintenance of the host strain that allows the effective and rapid introduction of expression vectors by transformation and selection with temperature. We demonstrate the usefulness of this system for the production of two recombinant proteins in E. coli, one selected to be produced as a soluble cytosolic protein and the other selected to be produced as an insoluble protein forming inclusion bodies. We further demonstrate the efficient overexpression of the cholera toxin B subunit (CTB) in V. cholerae and the secretion of the assembled pentameric protein product into the growth medium.

## RESULTS

### *lgt*-deleted mutant of E. coli strain BL21.

*lgt*-deleted strain MMS1742 was constructed and used to generate derivatives that expressed different recombinant proteins from plasmids maintained by the complementation of the chromosomal deletion of *lgt*, as described in Materials and Methods. Essentially, the *lgt* gene was removed from the chromosome of E. coli BL21 and complemented with the corresponding gene from V. cholerae harbored on either a temperature-sensitive maintenance plasmid that also conferred ampicillin resistance or a temperature-insensitive expression vector without any antibiotic selection markers.

### Expression of recombinant proteins in Δ*lgt* strains of E. coli (MMS1742).

**FIG 1:**
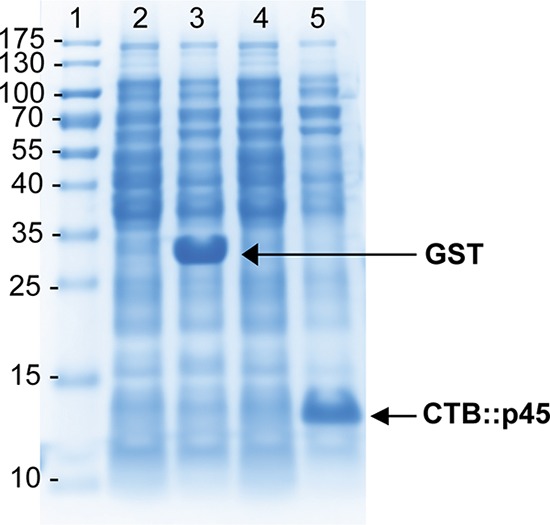
High-level expression of recombinant proteins in *lgt*-deleted strains. Shown is an SDS-PAGE gel of protein lysates of the E. coli BL21-derived *lgt*-deleted strain MMS1742 carrying expression plasmids expressing different recombinant proteins. Lanes 2 and 4 show the respective strains after culturing under noninducing conditions. Cultures were induced by the addition of IPTG. Lane 3 shows the expression of soluble recombinant sj26GST in strain MMS1808 (MMS1742/pMT-sj26GST/lgtVc), and lane 5 shows the expression of recombinant CTB::p45, which is insoluble and produced as inclusion bodies in strain MMS1762 (MMS1742/pMT-CTB::p45/lgtVc).

In order to demonstrate the capacity of the generated expression vectors to mediate recombinant protein expression, the soluble and biologically active protein sj26GST and CTB::p45, a protein fusion of the cholera toxin B subunit and a peptide derived from human low-density lipoprotein (LDL) that is produced as insoluble cytoplasmic inclusion bodies, were both expressed in MMS1742 harboring plasmids pMT-Sj26GST/lgtVc (strain MMS1808) and pMT-CTB::p45/lgtVc (strain MMS1762), respectively. Both proteins were expressed from the *tac* promoter under the control of the LacI repressor in small-scale cultures, and expression was induced by the addition of isopropyl-β-d-thiogalactopyranoside (IPTG) to the growth medium, as described in Materials and Methods. In order to compare induced and noninduced cultures, duplicate cultures of each strain were grown, and IPTG was added to only one of them. The remaining cultures were used as noninduced controls. The results are shown in Fig. 1. It can be seen that both proteins were well expressed when cultures were induced with IPTG.

### Stability assessment.

To assess the stability of plasmids maintained by the complementation of a chromosomal *lgt* deletion, *dmpB*, a gene encoding catechol 2,3-dioxigenase (C23O) (14), was cloned into the expression vector pMT-CTB::p45/lgtVc. The plasmid was electroporated into E. coli Δ*lgt* strain MMS1742. The resulting clone, MMS1766 (MMS1742/pMT-C23O/lgtVc), was passaged in liquid culture (Luria-Bertani [LB] broth [5 ml]) and plated out to single colonies by spreading serial dilutions onto LB agar plates after 5 and 40 generations. On each occasion, 100 single colonies were patched onto LB agar plates supplemented with 1 mM IPTG in order to induce C23O expression. All the colonies picked expressed C23O, as detected by spraying the plates with a 1-mg/ml solution of catechol, indicating 100% retention of the plasmid in the absence of any selection after both 5 and 40 generations. C23O converts catechol to 2-hydroxymuconic semialdehyde, which is bright yellow (plasmid construction is shown in Fig. S1 in the supplemental material).

### Comparison of growth and expression between wild-type strain BL21 and strain MMS1742 carrying the pMT-CTB::p45/lgtVc plasmid.

To investigate whether complementation of a chromosomal *lgt* gene lesion for plasmid maintenance has a metabolic cost or otherwise affects the growth of the strains in comparison with comparable clones maintained by antibiotic resistance markers, the growth of strain MMS1762 (MMS1742 carrying recombinant plasmid pMT-CTB::p45/lgtVc) was compared to that of *lgt^+^* parental strain BL21 carrying the equivalent plasmid maintained by ampicillin (Amp) selection (MMS1089).

### Growth curve.

**FIG 2:**
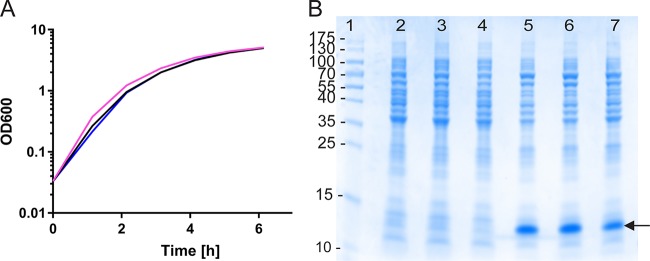
Comparable growth and protein expression levels in an E. coli Δ*lgt* strain compared with the wild-type strain carrying a conventionally maintained plasmid. (A) Duplicate growth curves of an E. coli BL21 Δ*lgt* Tn*5* (kan) strain MMS1742 derivative carrying pMT-CTB::p45/lgtVc (pink and blue lines) and wild-type strain E. coli BL21 carrying the equivalent plasmid pML-CTB::p45 maintained by Amp resistance (black line). (B) SDS-PAGE showing expression comparisons of the same strains grown under inducing (lanes 5 to 7) and noninducing (lanes 2 to 4) conditions. Lanes 2 and 5 and lanes 3 and 6 show protein from duplicate cultures of the MMS1742 derivatives grown under inducing and noninducing conditions, respectively, whereas lanes 4 and 7 show the parental BL21 strain carrying plasmid pML-CTB::p45 grown under inducing and noninducing conditions, respectively. The arrow indicates the recombinant protein.

Five-milliliter cultures of each strain were grown in 25-ml Lowenstein bottles in LB broth (supplemented with Amp when appropriate) overnight at 37°C with shaking. The optical density at 600 nm (OD_600_) of the starter cultures grown overnight was then measured and adjusted so that 25-ml LB cultures could be inoculated with the same starting inoculum (1:100 dilution of the adjusted inoculating cultures). The resulting 25-ml cultures were grown in 250-ml Erlenmeyer flasks at 37°C at 200 rpm, and the OD_600_ was measured every hour for 6 h. It could be seen that there was no significant difference in the growth rates or the final optical densities obtained with the corresponding wild-type strain (Fig. 2A).

### Expression test.

Five-milliliter cultures of each strain were grown overnight and used to inoculate duplicate 25-ml LB cultures. After 2 h, the expression of the recombinant protein was induced in one of each of the duplicates by the addition of IPTG to a final concentration of 1 mM, and cultures were incubated for a further 3 h at 37°C with shaking (200 rpm). The second cultures were retained as noninduced controls. After 3 h, the presence of inclusion bodies was confirmed by phase-contrast microscopy of the induced cultures. Equal numbers of cells from each culture were resuspended in 50 mM Tris-HCl (pH 7.5) and disrupted by sonication (1 min at 60% amplitude with 2-s pulses). Five microliters of each sample (induced and noninduced) in denaturing and reducing buffer was loaded into an SDS-PAGE gel (Fig. 2B), and the total protein, represented by the recombinant product measured by densitometry analysis, showed comparable expression levels.

The strains carrying plasmids for the recombinant expression of a CTB fusion protein under the same batch growth conditions showed comparable protein expression levels regardless of whether they were maintained by antibiotic selection or *lgt* complementation, indicating that the *lgt*-deleted strain carrying the plasmid is not affected by the deletion. This was also true of strains expressing sj26GST, which is expressed in the cytoplasm as a soluble protein (results not shown).

### Purification and biological activity of recombinant proteins expressed in *lgt*-deleted strains.

**FIG 3:**
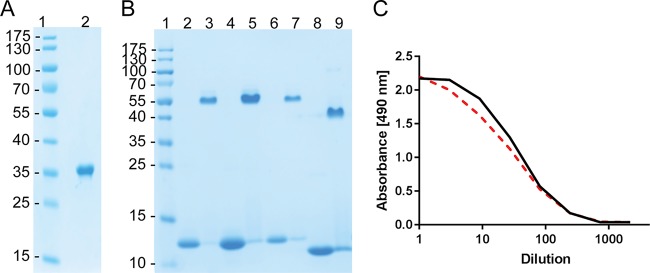
Purification and demonstration of the biological activity of recombinant proteins expressed as soluble cytoplasmic or inclusion body proteins in E. coli. (A) SDS-PAGE analysis following purification of sj26GST from the cytosol of strain MMS1808 (MMS1742/pMT-sj26GST/lgtVc). Lane 2 shows the GST protein after binding to a GST Hi-trap column and elution with 10 mM reduced glutathione. (B and C) Purification of CTB::p45 from inclusion bodies (B) and determination of biological activity by a GM1 ELISA (C). (B) Coomassie-stained SDS-PAGE gel showing purified proteins run under denaturing conditions (lanes 2, 4, 6, and 8) and nondenaturing conditions (lanes 3, 5, 7, and 9). Lanes 2 through 5 show CTB::p45 expressed from E. coli Δ*lgt* strain MMS1762. Lanes 6 and 7 show CTB::p45 expressed from BL21 carrying a conventional plasmid maintained by antibiotic selection (strain MMS1089). Lanes 8 and 9 show an rCTB standard. (C) GM1 ELISA of CTB::p45 produced from MMS1762 (black line) and MMS1089 (broken red line) after reassembly and purification. The presented data represent results from duplicate assays. The starting concentration of each protein was 0.5 μg/ml. The assay was performed as described in Materials and Methods.

In order to confirm the integrity and biological activity of the recombinant proteins produced from vectors maintained by *lgt* complementation in E. coli, both glutathione *S*-transferase (sj26GST), an active soluble protein produced in the cytoplasm (15), and CTB::p45, a cholera toxin B subunit fusion protein that is produced as insoluble inclusion bodies that must be isolated and reassembled *in vitro*, were expressed from the *tac* promoter in an *lgt*-maintained expression vector. The results of the purification of these proteins are shown in Fig. 3. These results demonstrate that GST can be purified by affinity chromatography based on reduced glutathione, which also indicates that the expressed protein has its expected biological activity (Fig. 3A). In the case of CTB::p45, the protein was successfully reassembled into a pentameric fusion protein as demonstrated by SDS-PAGE under nonreducing and reducing conditions (Fig. 3B). Binding to GM1 ganglioside in an enzyme-linked immunosorbent assay (ELISA) confirmed both that protein structure was restored, since the LT39 antibody used is specific for the pentameric form, and that the receptor binding properties of CTB were retained (Fig. 3C).

### Parallel construction of *lgt*-deleted Vibrio cholerae and production of recombinant CTB.

In the case of V. cholerae, the starting strain was JS1569, since derivatives of this strain are currently used for the industrial production of CTB. The V. cholerae derivative was generated by using an identical strategy in which the complementing *lgt* gene was derived from E. coli. All primers and plasmids used for the construction of V. cholerae strains MMS1663 [JS1569; Δ*lgt* pMT-lgtEc(ts)] and MMS1692 [JS1569; Δ*lgt* pMT-ctxB/lgtEc(ts)] are shown in Tables 1 and 2. Sequences and schematic representations of the plasmid constructs are shown in Section S2 in the supplemental material.

**TABLE 1:** Bacterial strains and plasmids used to generate V. cholerae Δ*lgt* strain MMS1633 and its derivative MM1692 carrying an expression vector for the production of recombinant CTB

Strain or plasmid	Phenotype or genotype	Reference(s) or source
Vibrio cholerae strains		
Cairo 50	Clinical isolate; serogroup O1 classical Ogawa	24
JS1569	Serogroup O1 classical V. cholerae; Δ*ctxA* Rif^r^	25
MMS1588	V. cholerae JS1569/pMT-suicide1-ΔlgtVc-Km carrying the temp-sensitive pMT-lgtEc(ts) plasmid	This study
MMS1589	Δ*lgt* Tn*5* (Km^r^) derivative of JS1569 carrying the temp-sensitive pMT-lgtEc(ts) plasmid	This study
MMS1633	MMS1589 in which the Km^r^ gene has been removed	This study
MMS1692	MMS1633 carrying CTB expression plasmid pMT-CTB/lgtEc in place of pMT-lgtEc(ts)	This study
Plasmids		
pMT-ssB	R6K-based suicide vector carrying the *sacB* gene from Bacillus subtilis	3, 4
pMT-ssB-ΔlgtVc	pMT-ssB carrying the deleted V. cholerae *lgt* gene (Δ*lgtVc*)	This study
pMT-ssB-ΔlgtVc/Km^r^	pMT-ssB carrying the deleted V. cholerae *lgt* gene and kanamycin gene (Km^r^)	This study
pBC loxP/Km	Used as a source of the *loxP*-flanked Km^r^ gene; confers Cm^r^ and Km^r^	This study
pMT-lgtEc(ts)	pSC101-derived temp-sensitive plasmid carrying the E. coli-derived *lgt* gene	This study
pMT-cre	pBR322-derived plasmid encoding the Cre recombinase expressed from the *tac* promoter under the control of the *lacI*^q^ repressor	This study
pML-LCTBtac	Expression plasmid derived from pAFtac1 and carrying *ctxB*; confers Amp^r^	M. Lebens, unpublished data
pMT-rCTB/lgtEc	pMT plasmid carrying recombinant CTB and the E. coli-derived *lgt* gene	This study

**TABLE 2:**
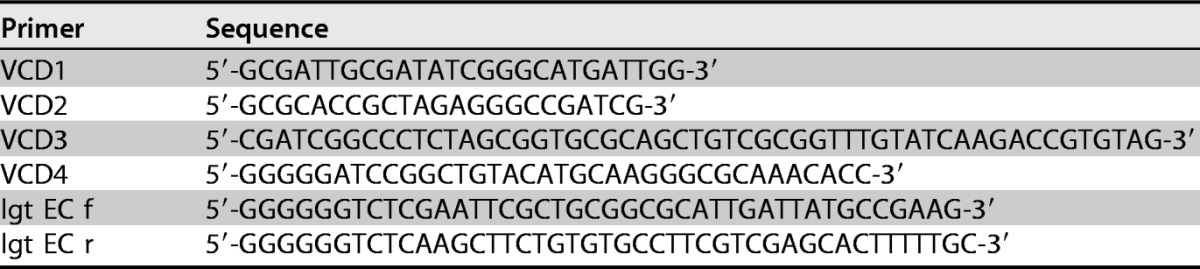
Primers used in this study in association with the construction of V. cholerae Δ*lgt* strain MMS1633

**FIG 4:**
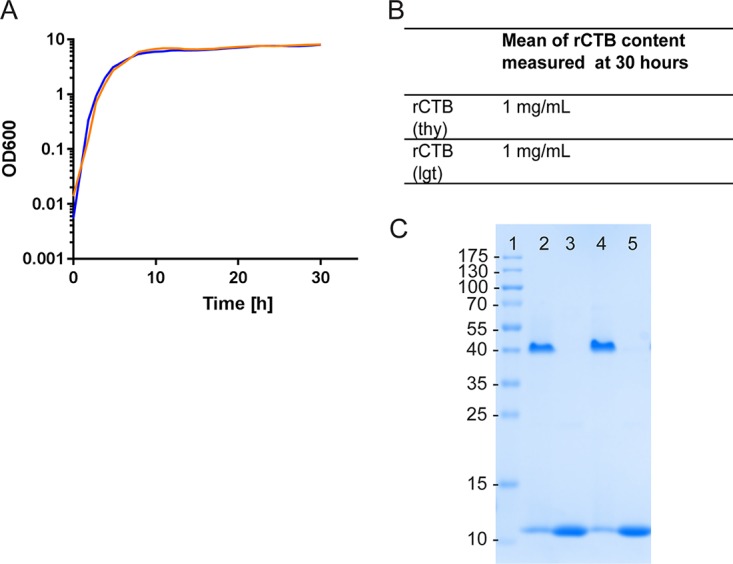
Comparable growth and expression of rCTB in different V. cholerae strains. (A) Growth curves of MS1012 expressing rCTB from a plasmid maintained by complementation of the *thyA* gene and MMS1692 expressing rCTB from a plasmid maintained by *lgt* complementation. (B) rCTB production in milligrams per milliliter, measured by a GM1 ELISA after 30 h. (C) Coomassie-stained SDS-PAGE gel showing rCTB in native (pentameric) and denatured forms from MMS1692 (lanes 2 and 3, respectively) and MMS1012 (lanes 4 and 5, respectively).

Recombinant CTB (rCTB) was produced from MMS1692. This is a soluble protein that is secreted and purified from the growth medium. The results of this purification are shown in Fig. 4 and demonstrate once again that the isolated protein retains its pentameric form and can bind to GM1 and react with LT39 antibody in a GM1 ELISA in a manner identical to that shown with rCTB produced from more conventional plasmids and furthermore showed equivalent productivity when grown in a 3-liter fermenter.

## DISCUSSION

In this paper, we describe the development of a novel method for maintaining plasmids without the need for antibiotic selection. This strategy is based on the use of strains from which an essential gene, *lgt*, has been deleted. Unlike most other systems that have been described, the removal of *lgt* from the E. coli strain under normal circumstances would be lethal irrespective of the growth conditions. Once the deletion is made, the cells are dependent upon a temperature-sensitive plasmid for survival. The replacement of this unstable plasmid with a temperature-insensitive plasmid is achieved by transformation and subsequent selection by growth at a nonpermissive temperature for the unstable plasmid. We have demonstrated the usefulness of this system by generating expression plasmids for the production of two recombinant proteins in E. coli, one (GST) chosen as a representative of a soluble cytoplasmic protein and another (CTB::p45 fusion protein) as an example of a protein expressed as inclusion bodies.

We have demonstrated extreme stability conferred on plasmids by *lgt* complementation in the absence of any antibiotic selection over a protracted period. In contrast, the corresponding plasmid still carrying an antibiotic selection marker in parental strain BL21 is highly unstable and rapidly lost in the absence of antibiotic selection (results not shown). Indeed, we made use of this inherent instability to select clones that have lost the pMT-Cre plasmid following the removal of the chromosomal Km^r^ gene during the construction of strains MMS1742 (E. coli Δ*lgt*) and MMS1663 (V. cholerae Δ*lgt*).

Hagg et al. (13) used a similar strategy with the *infA* gene, which was deleted from an E. coli strain and then complemented with the same gene residing on a plasmid. However, the system described in the present paper has important advantages. The gene complementing the deleted *lgt* gene is derived from V. cholerae and is therefore not homologous to the E. coli gene, even though it fulfills the same function. This made the strain construction strategy much less complicated since the only target for mutagenesis was located in the chromosome, and following the deletion of the gene, there was little chance for reversion through recombination. The V. cholerae-derived *lgt* gene is expressed from its natural promoter, and we could therefore construct expression vectors based on the *tac* promoter placed under the control of the *lacI*^q^ repressor gene. Constitutive expression could also be achieved by the removal of *lacI*^q^ from the plasmid by a simple digestion-and-religation procedure (results not shown). The production of CTB in V. cholerae is an example of constitutive expression from the *tac* promoter. It is perhaps noteworthy that the use of the operator repressor titration strategy (12) makes the use of such *lac*-derived expression systems impossible. Although we have demonstrated the use of IPTG-inducible expression here, we are confident that this system can be used in conjunction with vectors carrying alternative promoters, and vectors into which any promoter can be inserted by either *cre*-mediated recombination or ligation of restriction fragments are under construction.

Importantly, the growth of the *lgt*-deleted strains was not compromised in comparison with the parental strains, and the plasmids have proven to be extremely stable without the need for specialized media or supplementation with antibiotics. Likewise, the proteins expressed in *lgt*-deleted strains are functionally active, as shown by the activity based on the purification of GST, the expression and enzymatic activity of C23O in the plasmid stability test, and the pentamer assembly and GM1 receptor binding of both CTB::p45 and native CTB. Furthermore, the final yield of the recombinant protein was also fully comparable with that produced by a more conventional industrially used production plasmid.

Since the *lgt* gene is essential in all Gram-negative bacteria, it is quite possible to use this strategy for the production of recombinant proteins in other species. Thus, in the present study, in parallel with the E. coli system, we constructed a V. cholerae strain with the same lesion and demonstrated its usefulness for the high-yield production of CTB, which is an important component of both licensed (16) and newly developed (17) oral cholera vaccines. In this context, we have also demonstrated the scalability of this process by also successfully producing CTB in a 3-liter fermenter, and supported by our previous experience of successfully going from 3-liter fermentation to the industrial production of CTB on a 500-liter scale (our unpublished results), we see no reason why the process cannot be scaled up further in this system also.

In conclusion, the novel *lgt*-based selection system described here has proven to be extremely efficient and versatile and free from any restrictions imposed by the need for special growth media or temperatures. This system is therefore also a good candidate for use in the production of recombinant proteins in industrial-scale bacterial cultures. This is currently under investigation for both E. coli and V. cholerae strains generated in this paper.

## MATERIALS AND METHODS

### Bacterial strains and plasmids.

The plasmids and bacterial strains used in the present study are shown in Table 3. Unless stated otherwise, strains were maintained on LB or M9 minimal salts agar plates supplemented when necessary with appropriate antibiotics (chloramphenicol [Cm] at 12.5 μg/ml, Amp at 100 μg/ml, and kanamycin [Km] at 50 μg/ml) and stored in a 17% glycerol stock solution at −80°C. Liquid cultures were grown at 37°C in rotary shakers at 180 rpm unless otherwise specified. Specifically, strains carrying plasmids based on the temperature-sensitive pSC101 replicon were grown at 30°C.

**TABLE 3:** Bacterial strains and plasmids used to generate E. coli Δ*lgt* strain MMS1742 and its derivatives carrying expression vectors for the production of recombinant proteins

Strain or plasmid	Phenotype or genotype	Reference(s) or source
Strains		
E. coli		
S17-1	Tp^r^ Sm^r^ *recA thi pro hsdR^+^* RP4::2-Tc::Mu::Km Tn*7* λ*pir*	26
BL21	F^−^ *ompT* (r_B_^−^ m_B_^−^)	27
MMS1709	S17-1/pMT-ssB-ΔlgtEc-Km	This study
MMS1713	BL21/pMT-lgtVc(ts)	This study
MMS1716	Δ*lgt* Tn*5* (Km^r^) derivative of BL21 carrying temp-sensitive plasmid pMT-lgtVc(ts) (MMS1713)	This study
MMS1742	Δ*lgt* derivative of MMS1716 in which the Km^r^ gene has been removed	This study
MMS1089	BL21/pML-CTB::p45	M. Lebens, unpublished data
MMS1762	MMS1742 carrying pMT-CTB::p45/lgtVc in place of pMT-lgtVc(ts)	This study
MMS1097	BL21/pML-GST	
MMS1808	MMS1742 carrying pMT-sj26GST/lgtVc in place of pMT-lgtVc(ts)	This study
M317	N99cI^+^/pML-C23Oλ under the control of the **λ**pL promoter (source of *dmpB*)	Unpublished
MMS1766	MMS1742 carrying pMT-C23O/lgtVc in place of pMT-lgtVc(ts)	This study
Vibrio cholerae Cairo 50	Clinical isolate; serogroup O1 classical Ogawa	24
Plasmids		
pAFtac1	Expression vector for cloning and expression of recombinant proteins from the *tac* promoter; confers Amp^r^	19
pMT-ssB	R6K-based suicide vector carrying the *sacB* gene from B. subtilis; confers Cm^r^	3, 4
pMT-ssB-ΔlgtEc	pMT-ssB carrying the deleted E. coli *lgt* gene (Δ*lgtEc*)	This study
pMT-ssB-ΔlgtEc/Km^r^	pMT-ssB carrying the deleted E. coli *lgt* gene and the kanamycin resistance gene from Tn*5* (Km^r^)	This study
pBC loxP/Km	Used as a source of the *loxP*-flanked Km^r^ gene; confers Cm^r^ and Km^r^	This study
pMT-lgtVc(ts)	pSC101-derived temp-sensitive plasmid carrying the V. cholerae-derived *lgt* gene	This study
pMT-cre	pBR322-derived plasmid encoding the Cre recombinase expressed from the *tac* promoter under the control of the LacI repressor expressed from the plasmid-borne *lacI*^q^ gene; confers Cm^r^	This study
pML-CTB::p45	Plasmid carrying the CTB::p45 fusion protein expressed from the *tac* promoter under the control of the LacI repressor expressed from the plasmid-borne *lacI*^q^ gene; confers Amp^r^	M. Lebens, unpublished data
pMT-CTB::p45/lgtVc	Plasmid carrying the CTB::p45 fusion protein expressed from the *tac* promoter under the control of the LacI repressor expressed from the plasmid-borne *lacI*^q^ gene; carries the V. cholerae-derived *lgt* gene	This study
pML-sj26GST	Plasmid carrying the GST protein; confers Amp^r^	M. Lebens, unpublished data
pMT-sj26GST/lgtVc	Plasmid carrying the GST protein and the V. cholerae-derived *lgt* gene	This study
pML-C23Oλ	Plasmid carrying C23O	M. Lebens, unpublished data
pMT-C23O/lgtVc	Plasmid carrying C23O and the V. cholerae-derived *lgt* gene	This study

### DNA manipulation. (i) Chromosomal template DNA for PCR.

Chromosomal DNA from E. coli strain BL21 or V. cholerae strain JS1569 was obtained by suspending 3 to 5 colonies from a fresh LB agar plate in 200 μl water and boiling the colonies in a water bath for 5 min. Cell debris was removed by centrifugation, and 10 μl of the resulting supernatant was used as the template in 50-μl amplification reaction mixtures.

### (ii) Enzymes and reagents.

Enzymes, buffers, and deoxynucleotide mix solutions for restriction digestion of DNA, DNA modification, and PCR as well as reagents for agarose gel electrophoresis were all obtained from Thermo Fisher Scientific (Waltham, MA, USA) and used according to the manufacturer's instructions. Plasmids were prepared by using the ZR Plasmid Miniprep-classic kit (Zymo Research Corp., Irvine CA, USA). DNA products from PCRs and restriction analyses were analyzed by agarose gel electrophoresis using 1% agarose gels in 1× Tris-acetate-EDTA (TAE) buffer. All DNA sequencing of PCR fragments and plasmids was performed by Eurofins Genomics (Ebersberg, Germany).

### (iii) PCR and primerless PCR.

Primers used for the amplification of chromosomal DNA and subsequent primerless PCR were synthesized by Eurofins Genomics and are shown in Table 4.

**TABLE 4:**
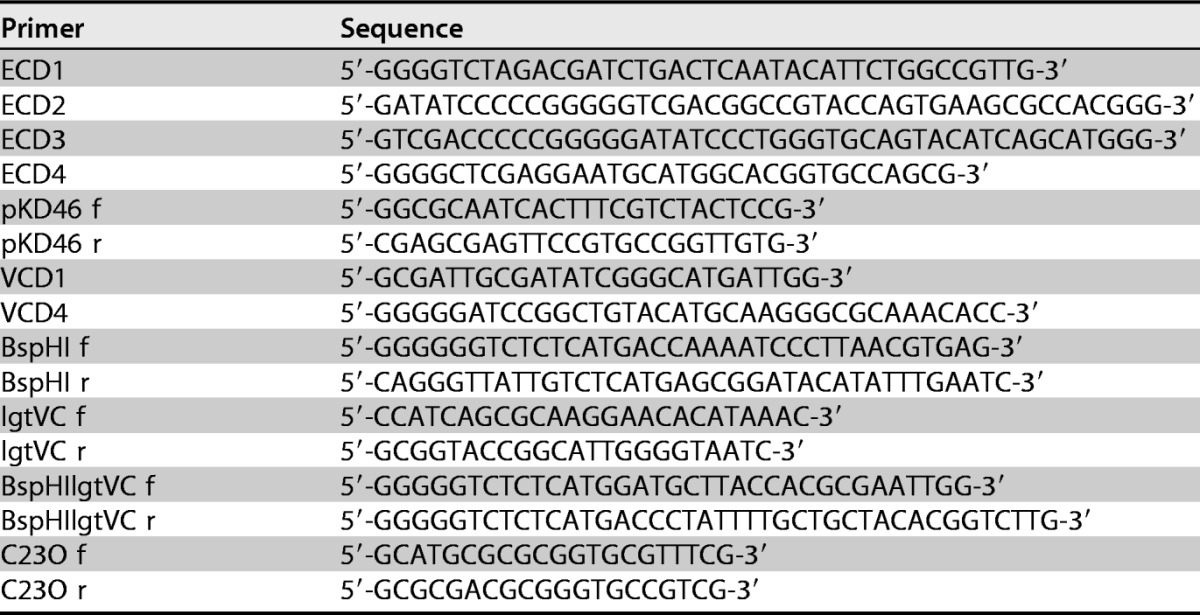
PCR primers used in this study in association with the construction of E. coli Δ*lgt* strain MMS1742 and associated plasmids

Conditions for standard PCR used for the amplification of chromosomal DNA fragments and primerless PCR used to fuse two amplified fragments together were described previously (16).

### (iv) Gel documentation.

Agarose gels were stained by using ethidium bromide and documented by using the EZ imager and Gel Doc software from Bio-Rad (Bio-Rad Laboratories AB, Solna, Sweden).

### (v) Electroporation and cell transformation.

Ligated DNA and/or plasmid preparations were electroporated into E. coli cells made competent according to the manufacturer's instructions, except that the cells were not first washed with HEPES buffer, by using a Gene Pulser II apparatus from Bio-Rad (Bio-Rad Laboratories AB, Solna, Sweden). Electrocompetent cells of V. cholerae were prepared according methods described previously by Lebens et al. (18). Transformants were selected on the basis of their antibiotic resistance and/or temperature selection (ability to grow at 39°C).

### Strain and plasmid construction.

The strain of E. coli used for the generation of *lgt* deletion mutants was BL21. This strain was chosen as it is widely used for the production of recombinant proteins.

### (i) Generation of a temperature-sensitive maintenance plasmid carrying native *lgt* from V. cholerae.

**FIG 5:**
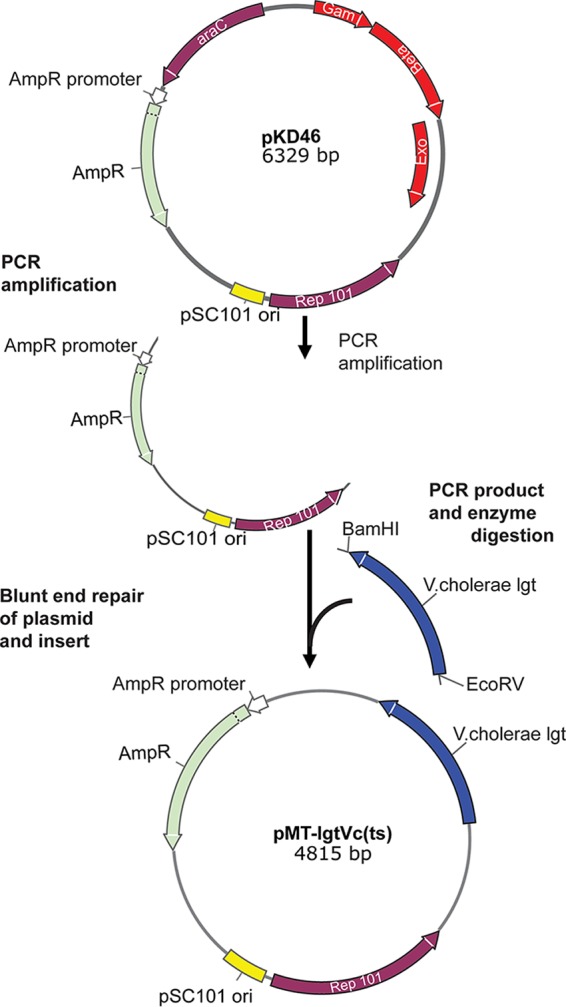
Cloning map of the pMT-lgtVc(ts) plasmid. pKD46 is derived from temperature-sensitive plasmid pSC101, which was amplified to obtain the replicon. The *lgt*-carrying region was amplified from V. cholerae with primers VCD1 and VCD4, which resulted in a DNA fragment flanked by BamHI and EcoRV restriction sites. After enzyme digestion, the insert and plasmid were blunt-end repaired and ligated. The ligated fragment was electroporated into electrocompetent BL21 cells.

In order to delete the *lgt* gene from the chromosome of any strain, it must first be present in the strain on a plasmid in order to complement the loss of the native gene. Accordingly, temperature-sensitive plasmids harboring the nonhomologous *lgt* gene cloned from either V. cholerae (for complementation in E. coli) or E. coli (for complementation in V. cholerae) were constructed. In the case of the plasmid complementing the mutation in E. coli, a 1,585-bp DNA fragment encoding the *lgt* gene was amplified from V. cholerae chromosomal template DNA by using primers VCD1 and VCD4 (see SEQ1 in Section S1 in the supplemental material). This fragment was digested with EcoRV and BamHI and blunt-end repaired with T4 DNA polymerase. The temperature-sensitive replicon derived from pSC101 was amplified from plasmid pKD46 by using primers pKD46f and pKD46r and also blunt-end repaired with T4 DNA polymerase. The two fragments were then ligated, and ligated DNA was used to transform E. coli strain XL1. Amp-resistant transformants were then screened for plasmids carrying the V. cholerae-derived *lgt* fragment by using PCR and restriction analysis of purified plasmid DNA. All incubations following the electroporation of competent cells were done at 30°C. The resulting plasmid was pMT-lgtVc(ts) (see SEQ2 in Section S1 in the supplemental material), which was used to transform E. coli strain BL21 (Fig. 5).

### (ii) Generation of an *lgt*-deleted strain of E. coli BL21.

The entire sequence of the fragment from BL21 harboring the *lgt* gene is shown in Section S1 in the supplemental material (SEQ3). Two fragments were amplified from chromosomal template DNA obtained from E. coli strain BL21 by using primer pairs ECD1/ECD2 and ECD3/ECD4, resulting in 650-bp and 595-bp fragments, respectively (see SEQ4 and SEQ5, respectively, in Section S1 in the supplemental material). These fragments were fused together by primerless PCR using primers ECD1 and ECD4 for the amplification of the final 1,225-bp *lgt*-deleted fragment (SEQ6 in Section S1). This fragment was digested with XbaI and XhoI and ligated into the suicide vector pMT-ssB digested with the same enzymes. Ligated DNA was then electroporated into E. coli strain S17-1, and transformants were selected on the basis of resistance to Cm. Plasmids were isolated from Cm-resistant transformants and screened for the insertion of the Δ*lgt* fragment by restriction analysis and PCR using primers ECD1 and ECD4. The resulting plasmid was pMT-ssB-ΔlgtEc (SEQ7 in Section S1).

**FIG 6:**
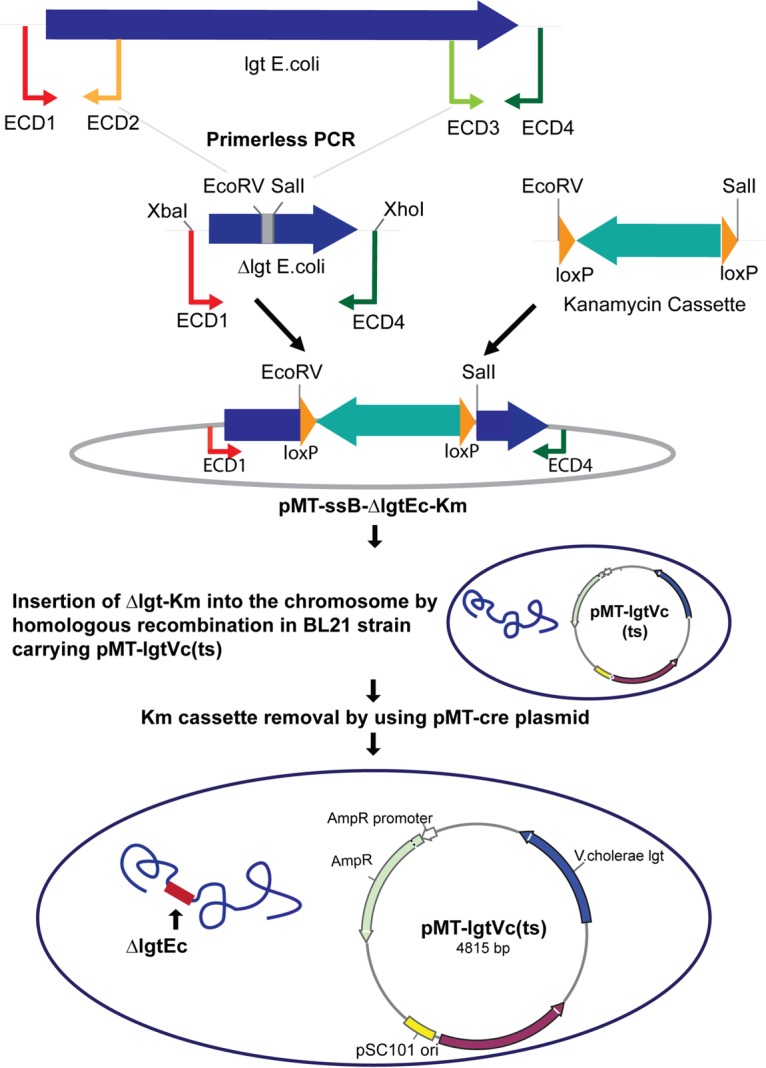
Schematic representation of the construction of Δ*lgt* strains of E. coli carrying temperature-sensitive plasmid pMT-lgtVc(ts). DNA fragments carrying a deletion of *lgt* were generated by primerless PCR and cloned into suicide plasmid pMT-ssB. A kanamycin resistance gene flanked by *loxP* sites was then inserted in place of the deleted *lgt* gene. The resulting plasmids were used to create deletions in the chromosomes of E. coli by allelic exchange. The kanamycin resistance gene was then removed with Cre recombinase. A temperature-sensitive (ts) plasmid carrying a nonhomologous *lgt* gene complements the chromosomal deletion, allowing the survival of the strain at 30°C.

A DNA fragment carrying the Km resistance gene from Tn*5* flanked by *loxP* sites was obtained from plasmid pBC loxP/Km (constructed in this laboratory) by digestion with SalI and EcoRV. DNA was ligated with pMT-ssB-ΔlgtEc digested with the same enzymes. Ligated DNA was electroporated into E. coli strain S17-1, and transformants were selected on the basis of their resistance to both Cm and Km. The plasmids carried by clones with the correct antibiotic resistances were isolated and analyzed by PCR and restriction analysis of purified plasmid DNA. The resulting plasmid was pMT-ssB-ΔlgtEc/Km^r^ (Fig. 6; see also SEQ8 in Section S1 in the supplemental material).

Plasmid pMT-ssB-ΔlgtEc/Km^r^ was then transferred into E. coli strain BL21 carrying temperature-sensitive plasmid pMT-lgtVc(ts) by transconjugation on LB plates at 30°C. Cells from these matings were grown on selective LB agar plates containing Amp and Km at 30°C. Colonies obtained from the selective plates were then grown on M9 minimal medium agar containing Amp and Km in order to completely eliminate any cells from the donor S17-1 strain. The resulting strain, which was resistant to kanamycin, ampicillin, and chloramphenicol, was grown in 5 ml liquid LB broth supplemented with Km and Amp only at 30°C overnight and then streaked out onto LB agar plates containing no salt but supplemented with sucrose to a final concentration of 6%. Single colonies from the sucrose plate were isolated and patched onto LB agar plates supplemented with Km and Amp and checked for sensitivity to Cm by duplicate plating onto LB agar plates containing Cm. Cm-sensitive colonies were confirmed to be BL21 derivatives by PCR to confirm the absence of the R6K *pir* gene present only in the S17-1 donor. Both the absence of the chromosomal *lgt* gene in the strains and its replacement with the Km resistance gene were confirmed by PCR using primers ECD1 and ECD4 followed by DNA sequencing. The resulting strain was called MMS1716 [BL21 Δ*lgt* Km^r^ (Tn*5*) pMT/lgtVc(ts)].

The Km resistance gene was removed from strain MMS1716 by Cre-mediated recombination. However, the commercially available plasmid for this procedure was based on the same replicon as that of pMT-lgtVc(ts) and could not be used. We therefore constructed a plasmid (pMT-Cre) based on the pBR322 origin of replication in which the *cre* gene was expressed from the *tac* promoter under the control of the *lacI*^q^ repressor. Furthermore, since pMT-lgtVc(ts) carries the *bla* gene conferring Amp resistance, we used the *cat* gene to confer Cm resistance. Thus, cells of MMS1716 were transformed with the pMT-Cre plasmid and selected on the basis of Cm resistance. Transformants were streaked out onto LB agar plates supplemented with Amp, Cm, and 1 mM IPTG (for the induction of Cre recombinase expression) and incubated at 30°C. Clones were patched in duplicate onto LB agar plates supplemented with either both Amp and Cm or Km alone and incubated at 30°C. Km-sensitive colonies were then streaked out onto LB agar plates supplemented with Amp and grown at 30°C overnight in order to allow the inherently unstable pMT-Cre plasmid to segregate out in the absence of selection. Single colonies were patched in duplicate onto LB agar plates supplemented with Amp or Cm and grown at 30°C. Amp-resistant colonies that were sensitive to both Km and Cm were analyzed by PCR amplification of chromosomal DNA with primers ECD1 and ECD4 and DNA sequencing to ensure that the Km resistance gene was removed (see SEQ9 in Section S1 in the supplemental material). The DNA was also amplified with primers lgtVC f and lgtVC r in order to confirm the presence of the complementing *lgt* gene from V. cholerae. The final strain was MMS1742 [BL21 Δ*lgt* pMT/lgtVc(ts)] (Fig. 6).

### (iii) Expression vectors and their construction and transformation into Δ*lgt* strains.

A feature of the system described here is its use for the expression of recombinant proteins without the need for antibiotic selection for the maintenance of plasmid expression vectors. This was achieved by using the temperature-sensitive nature of the complementation maintenance vector.

First, two expression vectors expressing two different recombinant proteins were constructed by using plasmids already constructed in this laboratory, pML-CTB::p45 and pML-sj26GST, both of which were derived from the general expression vector pAFtac1 (19). This was done by the replacement of the antibiotic resistance marker in each plasmid with the *lgt* gene from V. cholerae. The two plasmids were each amplified with primers BspHI f and BspHI r, which effectively resulted in amplified fragments carrying the entire replicons except for the antibiotic resistance marker (*bla*). The resulting fragments were digested with BspHI. The *lgt* gene was then amplified from V. cholerae strain JS1569 by using primers BspHIlgtVC f and BspHIlgtVC r, which resulted in an amplified fragment flanked with BspHI sites. This fragment was also digested with BspHI and ligated together with the amplified plasmid fragments from the two expression vectors.

**FIG 7:**
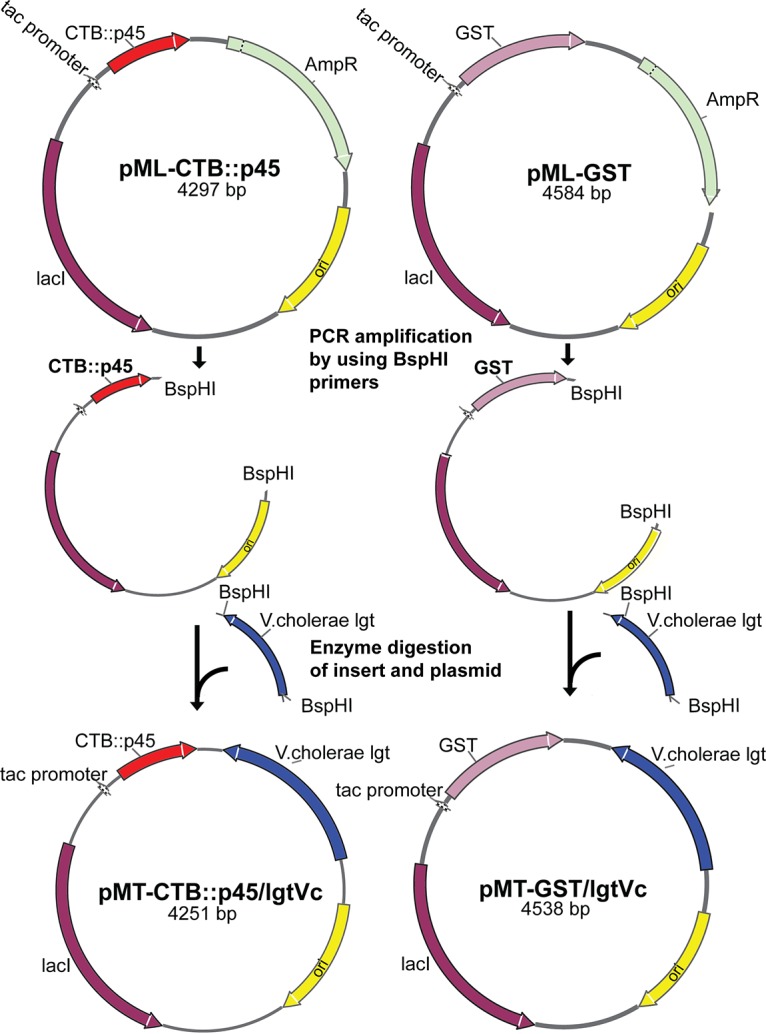
Cloning strategy for production of expression plasmids for use in E. coli strain MMS1742. Two plasmids already available in our laboratory were adapted to be used in Δ*lgt* strain MMS1742. The Amp resistance gene was removed by reverse PCR, which resulted in DNA fragments flanked by BspHI sites. A chromosomal fragment carrying the *lgt* gene was amplified from V. cholerae and also flanked by BspHI sites. The fragments were digested with BspHI and ligated. Ligated DNA was electroporated into electrocompetent cells of strain MMS1742 as described in the text and in the legend of Fig. 8.

**FIG 8:**
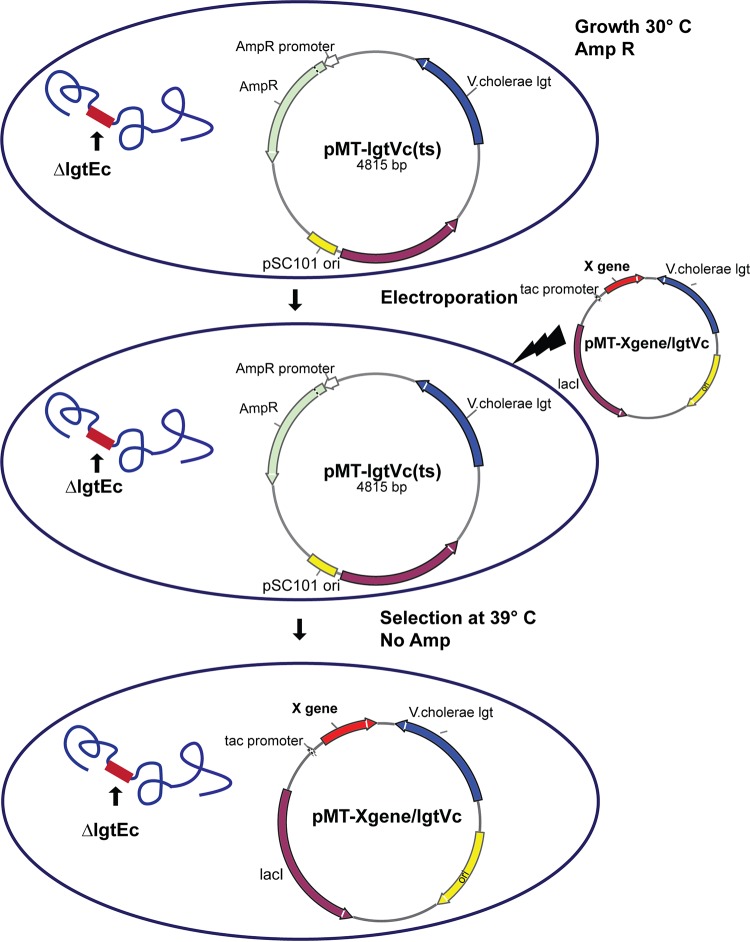
Replacement of the temperature-sensitive maintenance plasmid pMT-lgtVC(ts) in E. coli strain MMS1742 with an expression vector for production of recombinant plasmids. Cells are made electrocompetent and electroporated with a temperature-insensitive plasmid that can replicate at 39°C. The cells are plated out onto LB agar plates containing no antibiotics and incubated at the higher temperature. Colonies are tested for sensitivity to Amp.

Strain MMS1742 was maintained at 30°C and made electrocompetent. The ligated DNA from the expression vector constructs was electroporated into competent cells, which, following expression at 30°C, were plated out onto LB agar plates without any antibiotics and incubated at 39°C. At this nonpermissive temperature, the original strain cannot survive. Strains that had acquired the temperature-insensitive expression vector, however, would survive since the plasmids are retained at the higher temperature. Single colonies were picked from the transformation plates and tested for their sensitivity to Amp resulting from the loss of the temperature-sensitive plasmid. The presence of the expression vector in the resulting strains was confirmed by restriction analysis of isolated plasmids. The chromosomal deletion of the native *lgt* gene was also confirmed by PCR. The two plasmids were pMT-CTB::p45/lgtVc and pMT-Sj26GST/lgtVc. Plasmid maps are shown in Fig. 7, and the strategy for the introduction of expression vectors is shown in Fig. 8.

### Protein production and analysis. (i) Small- and medium-scale cultures for production of recombinant proteins.

*(a) Small scale*. Five-milliliter and 25-ml cultures of each strain were grown overnight at 37°C with shaking at 180 rpm and used to inoculate flasks (1:100 dilution) containing 25 ml and 500 ml fresh LB medium. The new cultures were incubated at 37°C with shaking (200 rpm), and after 2 h, the recombinant protein was induced by the addition of IPTG to a final concentration of 1 mM. Cultures were then incubated for a further 3 h before the bacteria were harvested by centrifugation.

*(b) Large scale*. Fifty-milliliter cultures were grown overnight in LB broth and used to inoculate a 5-liter bioreactor (Minifors; Infors HT, Switzerland) (1:100 dilution) containing 3 liters of Superbroth (SB). When the OD_600_ reached 0.6, the expression of the recombinant protein was induced by the addition of IPTG to a final concentration of 1 mM. Cultures were then incubated overnight at 37°C with aeration being set at 4 liters/min and with stirring at 600 rpm. The pH was set to 7.2 and maintained by the addition of 4 M HCl or 6.25 M NaOH. A 30% aqueous solution of Antifoam 204 (Sigma-Aldrich Co. LLC, USA) was added for foam control. Cultures were harvested after a total of 22 h.

When relevant, the presence of inclusion bodies was always confirmed by phase-contrast microscopy of the induced cultures.

### (ii) Protein purification.

Recombinant sj26GST was purified from soluble cell extracts produced by suspending the cells in 5 ml lysis buffer (50 mM Tris-HCl [pH 7.5], 1 mM EDTA, 1 mg/ml lysozyme) per g of cell pellet. The suspension was incubated for 15 min at room temperature before the addition of MgCl_2_ to a final concentration of 10 mM and DNase I (Roche) and kept for another 15 min at room temperature. Complete protease inhibitors (1 tablet per 50 ml of solution; Roche) were included during the lysis step. Cells were then disrupted by sonication (Bio-Rad Gene Pulser) on ice with 3 cycles of 5 min (2-s pulses at 60% amplitude). After centrifugation (6,000 × *g* for 30 min) to remove cell debris, the resulting supernatant was passed through a HiTrap reduced glutathione affinity column (GE Healthcare Life Sciences, England) connected to a Bio-Rad NGC chromatography system, according to the protocols provided by the manufacturer.

Recombinant CTB::p45 is a fusion protein in which CTB carries an additional peptide derived from human low-density lipoprotein (20) linked to its carboxyl terminus. It is expressed as inclusion bodies in E. coli and was purified essentially according to previously described methods (21). Following growth and induction of protein expression, cells were disrupted by lysozyme treatment followed by sonication as described above for the GST protein. Inclusion bodies were recovered by centrifugation at 3,000 × *g* for 30 min, extensively washed with 0.1% Triton X-114 and phosphate-buffered saline (PBS), and dissolved in 6.5 M urea. The proteins were reassembled by dialysis against sodium carbonate buffer (pH 9.0). Assembly was checked by SDS-PAGE, and the assembled proteins were purified by anion exchange chromatography using a Resource Q 6-ml anion exchange column (GE Healthcare Life Sciences, England) connected to a Bio-Rad NGC chromatography system. The protein was eluted with a linear gradient of 0 to 1 M NaCl in 50 mM carbonate buffer (pH 9).

Recombinant CTB was expressed as a native protein in V. cholerae. The product was secreted into the growth medium from which it was purified. Medium for the optimal production of CTB and methods for purification were described previously (22).

### (iii) SDS-PAGE.

SDS-PAGE was performed to analyze the expression of recombinant proteins prior to and after purification. Samples were analyzed by SDS-PAGE under reducing conditions, boiled and nonboiled, with the latter being used to visualize the assembled proteins where needed (CTB and CTB::p45).

Precast 14% Tris-glycine gels were obtained from Thermo Fisher and run on a Novex gel apparatus using commercially obtained SDS-PAGE Tris-glycine running buffer (Thermo Fisher). Gels were stained with Coomassie brilliant blue. Gels were recorded by using the same apparatus as the one used for agarose gels.

### (iv) GM1 ELISA.

A GM1 ELISA, which is based on the receptor-specific binding of protein to surface-coated GM1 ganglioside followed by detection of the bound protein by a specific antibody, was used to detect and quantify CTB and its derivatives and was performed as previously described (23).

## Supplementary Material

Supplemental material
