# Supplementary material for: A Novel Nonantibiotic, lgt-Based Selection System for Stable Maintenance of Expression Vectors in Escherichia coli and Vibrio cholerae
Source: Appl Environ Microbiol. 2018 Jan 31;84(4):e02143-17. doi: 10.1128/AEM.02143-17 (PMC5795084; doi:10.1128/AEM.02143-17)
Supplement: Supplemental material [file supp_84_4_e02143-17__index.html]

Supplemental material 

# A Novel Nonantibiotic, *lgt*-Based Selection System for Stable Maintenance of Expression Vectors in Escherichia coli and Vibrio cholerae

## Supplemental material

- Supplemental file 1 -

  Section 1 (sequence data for the construction of the *E. coli* strain BL21 *lgt*-deleted derivative MMS1742; cloning strategy of pMT-C23O/lgtVc plasmid used in the stability test in *E. coli* strain MMS1742 [Fig. S1]); section 2 [sequence data from the construction of the *V. cholerae* strain JS1569 *lgt*-deleted derivative MMS1663; construction of the maintenance plasmid pMT-lgtEc(ts) plasmid (Fig. S2); summary of the construction of the CTB production plasmid pMT-CTB/lgtEc (Fig. S3)].

  PDF, 443K
