# Supplementary material for: A Novel Nonantibiotic, lgt-Based Selection System for Stable Maintenance of Expression Vectors in Escherichia coli and Vibrio cholerae
Source: Appl Environ Microbiol. 2018 Jan 31;84(4):e02143-17. doi: 10.1128/AEM.02143-17 (PMC5795084; doi:10.1128/AEM.02143-17)
Supplement: Supplemental material [file AEM.02143-17_zam004188330s1.pdf]

A novel non-antibiotic, *lgt*-based selection system for stable maintenance of expression vectors in *Escherichia coli* and *Vibrio cholerae*.

Manuela Terrinoni[1], Stefan L. Nordqvist[1], Susanne Källgård[1], Jan Holmgren[1], Michael Lebens[1#].

[1]Department of Microbiology and Immunology and University of Gothenburg Vaccine Research Institute (GUVAX), Institute of Biomedicine, Sahlgrenska Academy at University of Gothenburg, Box 435, SE-405 30 Gothenburg, Sweden.

Running head: Plasmid selection & maintenance without antibiotics.

#Address correspondence: Michael Lebens, Department of Microbiology and Immunology, Institute of Biomedicine, Sahlgrenska Academy, University of Gothenburg, Medicinaregatan 7A, 41190 Gothenburg, Sweden. Email: michael.lebens@microbio.gu.se .Telephone: +46 31 786 6236

Keywords: Plasmid maintenance, gram negative bacteria, essential genes, complementation.

SUPPLEMENTARY DATA SECTION 1

Sequence data for the construction of the *E. coli* strain BL21 *lgt*-deleted derivative MMS1742.

SEQ 1 - *lgt* gene amplified from *Vibrio cholerae* using primers VCD1 and VCD4 (underlined). The fragment was digested with EcoRV and BamHI, blunt end repaired and inserted into a temperature sensitive vector derived from pSC101 (see text and figure 5 in the main text).The coding region for *lgt* is highlighted.

```
   1 GCGATTGCGA TATCGGGCAT GATTGGTTTT ATTTTGCACG GTTATCAAGT GGAGAACTTG
  61 CCACAATACA GCCTTGGTTA TGTTTATTTA CCTGCATTGT TAGCGATTGC TACAACATCG
 121 ATGCTTACCA CGCGAATTGG CGCTAAACTT GCCACCCAAA TGCCAACAGC AAGGCTTAAG
 181 CGATTCTTTG CCATTTTTTT AATGTGCGTC GCTGTGACCA TGTTGTTCCA GTAATACTCA
 241 TTGTTTATAG AGAAGGTTTG TTATGCCTCA GGGTTATCTG CAGTTTCCCA ATATTGACCC
 301 CGTATTGTTT TCGATCGGCC CTCTAGCGGT GCGCTGGTAT GGCTTGATGT ATTTGGTGGG
 361 TTTCCTTTTT GCTATGTGGT TGGCCAATCG CCGAGCGGAT CGCGCGGGCA GTGGTTGGAC
 421 GCGTGAGCAA GTCTCTGACT TGTTATTCGC CGGCTTTTTA GGTGTAGTGA TCGGTGGCCG
 481 AGTTGGTTAT GTGATCTTCT ACAATTTTGA TCTGTTCCTT GCTGACCCTC TTTATTTATT
 541 CAAAGTGTGG ACTGGCGGCA TGTCCTTCCA CGGCGGCTTA TTGGGTGTGA TCACCGCCAT
 601 GTTCTGGTAT GCGCGTAAAA ACCAACGCAC CTTCTTTGGT GTGGCCGATT TTGTTGCCCC
 661 TTTAGTGCCA TTCGGTTTGG GGATGGGACG TATCGGTAAC TTTATGAATA GTGAACTTTG
 721 GGGACGAGTA ACGGATGTGC CTTGGGCTTT TGTATTCCCT AATGGTGGCC CACTGCCGCG
 781 CCATCCTTCA CAGCTTTATG AATTCGCCTT AGAAGGCGTG GTTCTGTTCT TTATTCTTAA
 841 TTGGTTTATT GGTAAACCTC GTCCGCTAGG CAGCGTATCC GGACTGTTTT TAGCTGGATA
 901 CGGTACATTC CGCTTCCTTG TGGAATACGT CCGTGAGCCA GATGCTCAGT TGGGTCTGTT
 961 TGGTGGCTTC ATTTCAATGG GGCAAATCCT CTCCTTACCT ATGGTGATCA TCGGTATTTT
1021 GATGATGGTT TGGTCTTACA AGCGCGGTTT GTATCAAGAC CGTGTAGCAG CAAAATAGGG
1081 TAGTTAGGTG AGACAGTATT TAGATCTTTG TCAGCGCATC GTCGATCAAG GTGTTTGGGT
1141 TGAAAATGAA CGAACGGGCA AGCGTTGTTT GACTGTGATT AATGCCGATT TGACCTACGA
1201 TGTGGGCAAC AATCAGTTTC CTCTAGTGAC TACACGCAAG AGTTTTTGGA AAGCTGCCGT
1261 AGCCGAGTTG CTCGGCTATA TTCGTGGTTA CGATAATGCG GCGGATTTTC GCCAATTAGG
1321 TACCAAAACC TGGGATGCTA ATGCCAATTT AAACCAAGCA TGGCTCAACA ATCCTTACCG
1381 TAAAGGTGAG GATGACATGG GACGCGTGTA TGGTGTTCAG GGTAGAGCTT GGGCTAAGCC
1441 TGATGGTGGT CATATTGACC AGTTGAAAAA GATTGTTGAT GATTTGAGCC GTGGCGTTGA
1501 TGACCGAGGT GAAATTCTTA ACTTCTACAA TCCGGGTGAA TTTCACATGG GGTGTTTGCG
1561 CCCTTGCATG TACAGCCGGA TCCCCC
```

SEQ 2- the entire replicon pMT-lgtVC(ts) for maintenance of lgt-deleted strains of *E. coli*.

The *V. cholerae*-derived *lgt* fragment is highlighted and the *lgt* coding region is underlined.

The primer regions used for amplification of the plasmid are also underlined.

```
   1 ATCGGGCATG ATTGGTTTTA TTTTGCACGG TTATCAAGTG GAGAACTTGC CACAATACAG
  61 CCTTGGTTAT GTTTATTTAC CTGCATTGTT AGCGATTGCT ACAACATCGA TGCTTACCAC
 121 GCGAATTGGC GCTAAACTTG CCACCCAAAT GCCAACAGCA AGGCTTAAGC GATTCTTTGC
 181 CATTTTTTTA ATGTGCGTCG CTGTGACCAT GTTGTTCCAG TAATACTCAT TGTTTATAGA
 241 GAAGGTTTGT TATGCCTCAG GGTTATCTGC AGTTTCCCAA TATTGACCCC GTATTGTTTT
 301 CGATCGGCCC TCTAGCGGTG CGCTGGTATG GCTTGATGTA TTTGGTGGGT TTCCTTTTTG
 361 CTATGTGGTT GGCCAATCGC CGAGCGGATC GCGCGGGCAG TGGTTGGACG CGTGAGCAAG
 421 TCTCTGACTT GTTATTCGCC GGCTTTTTAG GTGTAGTGAT CGGTGGCCGA GTTGGTTATG
 481 TGATCTTCTA CAATTTTGAT CTGTTCCTTG CTGACCCTCT TTATTTATTC AAAGTGTGGA
 541 CTGGCGGCAT GTCCTTCCAC GGCGGCTTAT TGGGTGTGAT CACCGCCATG TTCTGGTATG
 601 CGCGTAAAAA CCAACGCACC TTCTTTGGTG TGGCCGATTT TGTTGCCCCT TTAGTGCCAT
 661 TCGGTTTGGG GATGGGACGT ATCGGTAACT TTATGAATAG TGAACTTTGG GGACGAGTAA
 721 CGGATGTGCC TTGGGCTTTT GTATTCCCTA ATGGTGGCCC ACTGCCGCGC CATCCTTCAC
 781 AGCTTTATGA ATTCGCCTTA GAAGGCGTGG TTCTGTTCTT TATTCTTAAT TGGTTTATTG
 841 GTAAACCTCG TCCGCTAGGC AGCGTATCCG GACTGTTTTT AGCTGGATAC GGTACATTCC
 901 GCTTCCTTGT GGAATACGTC CGTGAGCCAG ATGCTCAGTT GGGTCTGTTT GGTGGCTTCA
 961 TTTCAATGGG GCAAATCCTC TCCTTACCTA TGGTGATCAT CGGTATTTTG ATGATGGTTT
1021 GGTCTTACAA GCGCGGTTTG TATCAAGACC GTGTAGCAGC AAAATAGGGT AGTTAGGTGA
1081 GACAGTATTT AGATCTTTGT CAGCGCATCG TCGATCAAGG TGTTTGGGTT GAAAATGAAC
1141 GAACGGGCAA GCGTTGTTTG ACTGTGATTA ATGCCGATTT GACCTACGAT GTGGGCAACA
1201 ATCAGTTTCC TCTAGTGACT ACACGCAAGA GTTTTTGGAA AGCTGCCGTA GCCGAGTTGC
1261 TCGGCTATAT TCGTGGTTAC GATAATGCGG CGGATTTTCG CCAATTAGGT ACCAAAACCT
1321 GGGATGCTAA TGCCAATTTA AACCAAGCAT GGCTCAACAA TCCTTACCGT AAAGGTGAGG
1381 ATGACATGGG ACGCGTGTAT GGTGTTCAGG GTAGAGCTTG GGCTAAGCCT GATGGTGGTC
1441 ATATTGACCA GTTGAAAAAG ATTGTTGATG ATTTGAGCCG TGGCGTTGAT GACCGAGGTG
1501 AAATTCTTAA CTTCTACAAT CCGGGTGAAT TTCACATGGG GTGTTTGCGC CCTTGCATGT
1561 ACAGCCGGAT CGCGAGTTCC GTGCCGGTTG TGAAGAAAAA GTGAATGATG TAGCCGTCAA
1621 GTTGTCATAA TAAATCGATG CAGGTGGCAC TTTTCGGGGA AATGTGCGCG GAACCCCTAT
1681 TTGTTTATTT TTCTAAATAC ATTCAAATAT GTATCCGCTC ATGAGACAAT AACCCTGATA
1741 AATGCTTCAA TAATATTGAA AAAGGAAGAG TATGAGTATT CAACATTTCC GTGTCGCCCT
1801 TATTCCCTTT TTTGCGGCAT TTTGCCTTCC TGTTTTTGCT CACCCAGAAA CGCTGGTGAA
1861 AGTAAAAGAT GCTGAAGATC AGTTGGGTGC ACGAGTGGGT TACATCGAAC TGGATCTCAA
1921 CAGCGGTAAG ATCCTTGAGA GTTTTCGCCC CGAAGAACGT TTTCCAATGA TGAGCACTTT
1981 TAAAGTTCTG CTATGTGGCG CGGTATTATC CCGTATTGAC GCCGGGCAAG AGCAACTCGG
2041 TCGCCGCATA CACTATTCTC AGAATGACTT GGTTGAGTAC TCACCAGTCA CAGAAAAGCA
2101 TCTTACGGAT GGCATGACAG TAAGAGAATT ATGCAGTGCT GCCATAACCA TGAGTGATAA
2161 CACTGCGGCC AACTTACTTC TGACAACGAT CGGAGGACCG AAGGAGCTAA CCGCTTTTTT
2221 GCACAACATG GGGGATCATG TAACTCGCCT TGATCGTTGG GAACCGGAGC TGAATGAAGC
2281 CATACCAAAC GACGAGCGTG ACACCACGAT GCCTGTAGCA ATGGCAACAA CGTTGCGCAA
2341 ACTATTAACT GGCGAACTAC TTACTCTAGC TTCCCGGCAA CAATTAATAG ACTGGATGGA
2401 GGCGGATAAA GTTGCAGGAC CACTTCTGCG CTCGGCCCTT CCGGCTGGCT GGTTTATTGC
2461 TGATAAATCT GGAGCCGGTG AGCGTGGGTC TCGCGGTATC ATTGCAGCAC TGGGGCCAGA
2521 TGGTAAGCCC TCCCGTATCG TAGTTATCTA CACGACGGGG AGTCAGGCAA CTATGGATGA
2581 ACGAAATAGA CAGATCGCTG AGATAGGTGC CTCACTGATT AAGCATTGGT AACTGTCAGA
2641 CCAAGTTTAC TCATATATAC TTTAGATTGA TTTAAAACTT CATTTTTAAT TTAAAAGGAT
2701 CTAGGTGAAG ATCCTTTTTG ATAATCTCAT GACCAAAATC CCTTAACGTG AGTTTTCGTT
2761 CCACTGAGCG TCAGACCCCG TTGATGATAC CGCTGCCTTA CTGGGTGCAT TAGCCAGTCT
2821 GAATGACCTG TCACGGGATA ATCCGAAGTG GTCAGACTGG AAAATCAGAG GGCAGGAACT
2881 GCTGAACAGC AAAAAGTCAG ATAGCACCAC ATAGCAGACC CGCCATAAAA CGCCCTGAGA
2941 AGCCCGTGAC GGGCTTTTCT TGTATTATGG GTAGTTTCCT TGCATGAATC CATAAAAGGC
3001 GCCTGTAGTG CCATTTACCC CCATTCACTG CCAGAGCCGT GAGCGCAGCG AACTGAATGT
3061 CACGAAAAAG ACAGCGACTC AGGTGCCTGA TGGTCGGAGA CAAAAGGAAT ATTCAGCGAT
3121 TTGCCCGATT GCGGCCGCAA CCGAGCTTGC GAGGGTGCTA CTTAAGCCTT TAGGGTTTTA
```

3181 AGGTCTGTTT TGTAGAGGAG CAAACAGCGT TTGCGACATC CTTTTGTAAT ACTGCGGAAC
3241 TGACTAAAGT AGTGAGTTAT ACACAGGGCT GGGATCTATT CTTTTTATCT TTTTTTATTC
3301 TTTCTTTATT CTATAAATTA TAACCACTTG AATATAAACA AAAAAAACAC ACAAAGGTCT
3361 AGCGGAATTT ACAGAGGGTC TAGCAGAATT TACAAGTTTT CCAGCAAAGG TCTAGCAGAA
3421 TTTACAGATA CCCACAACTC AAAGGAAAAG GACTAGTAAT TATCATTGAC TAGCCCATCT
3481 CAATTGGTAT AGTGATTAAA ATCACCTAGA CCAATTGAGA TGTATGTCTG AATTAGTTGT
3541 TTTCAAAGCA AATGAACTAG CGATTAGTCG CTATGACTTA ACGGAGCATG AAACCAAGCT
3601 AATTTTATGC TGTGTGGCAC TACTCAACCC CACGATTGAA AACCCTACAA GGAAAGAACG
3661 GACGGTATCG TTCACTTATA ACCAATACGC TCAGATGATG AACATCAGTA GGGAAAATGC
3721 TTATGGTGTA TTAGCTAAAG CAACCAGAGA GCTGATGACG AGAACTGTGG AAATCAGGAA
3781 TCCTTTGGTT AAAGGCTTTG AGATTTTCCA GTGGACAAAC TATGCCAAGT TCTCAAGCGA
3841 AAAATTAGAA TTAGTTTTTA GTGAAGAGAT ATTGCCTTAT CTTTTCCAGT TAAAAAAATT
3901 CATAAAATAT AATCTGGAAC ATGTTAAGTC TTTTGAAAAC AAATACTCTA TGAGGATTTA
3961 TGAGTGGTTA TTAAAAGAAC TAACACAAAA GAAAACTCAC AAGGCAAATA TAGAGATTAG
4021 CCTTGATGAA TTTAAGTTCA TGTTAATGCT TGAAAATAAC TACCATGAGT TTAAAAGGCT
4081 TAACCAATGG GTTTTGAAAC CAATAAGTAA AGATTTAAAC ACTTACAGCA ATATGAAATT
4141 GGTGGTTGAT AAGCGAGGCC GCCCGACTGA TACGTTGATT TTCCAAGTTG AACTAGATAG
4201 ACAAATGGAT CTCGTAACCG AACTTGAGAA CAACCAGATA AAAATGAATG GTGACAAAAT
4261 ACCAACAACC ATTACATCAG ATTCCTACCT ACGTAACGGA CTAAGAAAAA CACTACACGA
4321 TGCTTTAACT GCAAAAATTC AGCTCACCAG TTTTGAGGCA AAATTTTTGA GTGACATGCA
4381 AAGTAAGTAT GATCTCAATG GTTCGTTCTC ATGGCTCACG CAAAAACAAC GAACCACACT
4441 AGAGAACATA CTGGCTAAAT ACGGAAGGAT CTGAGGTTCT TATGGCTCTT GTATCTATCA
4501 GTGAAGCATC AAGACTAACA AACAAAAGTA GAACAACTGT TCACCGTTAC ATATCAAAGG
4561 GAAAACTGTC CATACCCATG GATTCTTCGT CTGTTTCTAC TGGTATTGGC ACAAACCTGA
4621 TTCCAATTTG AGCAAGGCTA TGTGCCATCT CGATACTCGT TCTTAACTCA ACAGAAGATG
4681 CTTTGTGCAT ACAGCCCCTC GTTTATTATT TATCTCCTCA GCCAGCCGCT GTGCTTTCAG
4741 TGGATTTCGG ATAACAGAAA GGCCGGGAAA TACCCAGCCT CGCTTTGTAA CGGAGTAGAC
4801 GAAAGTGATT GCGCC

SEQ3 - Native DNA encoding *lgt* in *E. coli* strain BL21 deleted region highlighted in blue.

The coding region for lgt is underlined.

```
   1 CGATCTGACT CAATACATTC TGGCCGTTGA TCGCAACAAT ACCCGGGTGG CGAACATTTA
  61 TGACAGTCTT CATCCTGCAA TGTTACGAGC TCTGGCGATG ATCGCCCGGG AAGCGGAAAT
 121 ACATGGAATC GATCTCCGTT TGTGCGGTGA AATGGCGGGC GATCCCATGT GCGTGGCAAT
 181 CCTCATTGGG CTTGGGTATC GCCATCTGTC TATGAACGGA CGTTCTGTAG CGCGGGCAAA
 241 ATACCTGCTG CGGCGCATTG ATTATGCCGA AGCAGAAAAT CTTGCGCAGC GTAGTCTGGA
 301 AGCGCAACTG GCGACCGAAG TTCGCCATCA GGTTGCAGCC TTTATGGAGC GTCGCGGCAT
 361 GGGCGGGCTG ATTCGCGGAG GGTTATAGCG CGGATCATAT ACATATCTTT TAACGGTATC
 421 CGGCAACCAG CCAGGTCCCC TTGTGCTATT ATTCGCACCT TTGGAGCGCC TGAAACCTGC
 481 GGCGCGCATT TCAATCGCTG TTCTCTTTCA GCGAAATAAC AAGAACTTGT GGTGACAGAT
 541 GACCAGTAGC TATCTGCATT TTCCGGAGTT TGATCCGGTC ATTTTCTCAA TAGGACCCGT
 601 GGCGCTTCAC TGGTACGGCC TGATGTATCT GGTGGGTTTC ATTTTTGCAA TGTGGCTGGC
 661 AACACGACGG GCGAATCGTC CGGGCAGCGG CTGGACCAAA AATGAAGTTG AAAACTTACT
 721 CTATGCGGGC TTCCTCGGCG TCTTCCTCGG GGGACGTATT GGTTATGTTC TGTTCTACAA
 781 TTTCCCGCAG TTTATGGCCG ATCCGCTGTA TCTGTTCCGT GTCTGGGACG GCGGCATGTC
 841 TTTCCACGGC GGCCTGATTG GCGTTATCGT GGTGATGATT ATCTTCGCCC GCCGTACTAA
 901 ACGTTCCTTC TTCCAGGTCT CTGATTTTAT CGCACCACTC ATTCCGTTTG GTCTTGGTGC
 961 CGGGCGTCTG GGCAACTTTA TTAACGGTGA ATTGTGGGGC CGCGTTGACC CGAACTTCCC
1021 GTTTGCCATG CTGTTCCCTG GCTCCCGTAC AGAAGATATT TTGCTGCTGC AAACCAACCC
1081 GCAGTGGCAA TCCATTTTCG ACACTTACGG TGTGCTGCCG CGCCACCCAT CACAGCTTTA
1141 CGAGCTGCTG CTGGAAGGTG TGGTGCTGTT TATTATCCTC AACCTGTATA TTCGTAAACC
1201 ACGCCCAATG GGAGCTGTCT CAGGTTTGTT CCTGATTGGT TACGGCGCGT TTCGCATCAT
1261 TGTTGAGTTT TTCCGCCAGC CCGACGCGCA GTTTACCGGT GCCTGGGTGC AGTACATCAG
1321 CATGGGGCAA ATTCTTTCCA TCCCGATGAT TGTCGCGGGT GTGATCATGA TGGTCTGGGC
1381 ATATCGTCGC AGCCCACAGC AACACGTTTC CTGAGGAACC ATGAAACAGT ATTTAGAACT
1441 GATGCAAAAA GTGCTCGACG AAGGCACACA GAAAAACGAC CGTACCGGAA CCGGAACGCT
1501 TTCCATTTTT GGTCATCAGA TGCGTTTTAA CCTGCAAGAT GGATTCCCGC TGGTGACAAC
1561 TAAACGTTGC CACCTGCGTT CCATCATCCA TGAACTGCTG TGGTTTCTGC AGGGCGACAC
1621 TAACATTGCT TATCTACACG AAAACAATGT CACCATCTGG GACGAATGGG CCGATGAAAA
1681 CGGCGACCTC GGGCCAGTGT ATGGTAAACA GTGGCGCGCC TGGCCAACGC CAGATGGTCG
1741 TCATATTGAC CAGATCACTA CGGTACTGAA CCAGCTGAAA AACGACCCGG ATTCGCGCCG
1801 CATTATTGTT TCAGCGTGGA ACGTAGGCGA ACTGGATAAA ATGGCGCTGG CACCGTGCCA
1861 TGCATTC
```

SEQ4 - Amplified DNA using primer pair ECD1/ECD2 (Primer sequences underlined). The coding region for *lgt* is highlighted

```
  1 GGGGTCTAGA CGATCTGACT CAATACATTC TGGCCGTTGA TCGCAACAAT ACCCGGGTGG
 61 CGAACATTTA TGACAGTCTT CATCCTGCAA TGTTACGAGC TCTGGCGATG ATCGCCCGGG
121 AAGCGGAAAT ACATGGAATC GATCTCCGTT TGTGCGGTGA AATGGCGGGC GATCCCATGT
181 GCGTGGCAAT CCTCATTGGG CTTGGGTATC GCCATCTGTC TATGAACGGA CGTTCTGTAG
241 CGCGGGCAAA ATACCTGCTG CGGCGCATTG ATTATGCCGA AGCAGAAAAT CTTGCGCAGC
301 GTAGTCTGGA AGCGCAACTG GCGACCGAAG TTCGCCATCA GGTTGCAGCC TTTATGGAGC
361 GTCGCGGCAT GGGCGGGCTG ATTCGCGGAG GGTTATAGCG CGGATCATAT ACATATCTTT
421 TAACGGTATC CGGCAACCAG CCAGGTCCCC TTGTGCTATT ATTCGCACCT TTGGAGCGCC
481 TGAAACCTGC GGCGCGCATT TCAATCGCTG TTCTCTTTCA GCGAAATAAC AAGAACTTGT
541 GGTGACAGAT GACCAGTAGC TATCTGCATT TTCCGGAGTT TGATCCGGTC ATTTTCTCAA
601 TAGGACCCGT GGCGCTTCAC TGGTACGGCC GTCGACCCCC GGGGGATATC
```

SEQ 5 - Amplified DNA using primer pair ECD3/ECD4 (primer sequences underlined). The cdoing region for *lgt* is highlighted

```
  1 GTCGACCCCC GGGGGATATC CCTGGGTGCA GTACATCAGC ATGGGGCAAA TTCTTTCCAT
 61 CCCGATGATT GTCGCGGGTG TGATCATGAT GGTCTGGGCA TATCGTCGCA GCCCACAGCA
121 ACACGTTTCC TGAGGAACCA TGAAACAGTA TTTAGAACTG ATGCAAAAAG TGCTCGACGA
181 AGGCACACAG AAAAACGACC GTACCGGAAC CGGAACGCTT TCCATTTTTG GTCATCAGAT
241 GCGTTTTAAC CTGCAAGATG GATTCCCGCT GGTGACAACT AAACGTTGCC ACCTGCGTTC
301 CATCATCCAT GAACTGCTGT GGTTTCTGCA GGGCGACACT AACATTGCTT ATCTACACGA
361 AAACAATGTC ACCATCTGGG ACGAATGGGC CGATGAAAAC GGCGACCTCG GGCCAGTGTA
421 TGGTAAACAG TGGCGCGCCT GGCCAACGCC AGATGGTCGT CATATTGACC AGATCACTAC
481 GGTACTGAAC CAGCTGAAAA ACGACCCGGA TTCGCGCCGC ATTATTGTTT CAGCGTGGAA
541 CGTAGGCGAA CTGGATAAAA TGGCGCTGGC ACCGTGCCAT GCATTCCTCG AGCCC
```

SEQ 6 - Amplified DNA following primerless PCR and amplification with primers ECD1 and ECD4 (underlined). Remaining lgt sequence is highlighted in blue and the linker replacing the coding region for *lgt* is highlighted in yellow.

```
  1 GGGGTCTAGA CGATCTGACT CAATACATTC TGGCCGTTGA TCGCAACAAT ACCCGGGTGG
 61 CGAACATTTA TGACAGTCTT CATCCTGCAA TGTTACGAGC TCTGGCGATG ATCGCCCGGG
121 AAGCGGAAAT ACATGGAATC GATCTCCGTT TGTGCGGTGA AATGGCGGGC GATCCCATGT
181 GCGTGGCAAT CCTCATTGGG CTTGGGTATC GCCATCTGTC TATGAACGGA CGTTCTGTAG
241 CGCGGGCAAA ATACCTGCTG CGGCGCATTG ATTATGCCGA AGCAGAAAAT CTTGCGCAGC
301 GTAGTCTGGA AGCGCAACTG GCGACCGAAG TTCGCCATCA GGTTGCAGCC TTTATGGAGC
361 GTCGCGGCAT GGGCGGGCTG ATTCGCGGAG GGTTATAGCG CGGATCATAT ACATATCTTT
421 TAACGGTATC CGGCAACCAG CCAGGTCCCC TTGTGCTATT ATTCGCACCT TTGGAGCGCC
481 TGAAACCTGC GGCGCGCATT TCAATCGCTG TTCTCTTTCA GCGAAATAAC AAGAACTTGT
541 GGTGACAGAT GACCAGTAGC TATCTGCATT TTCCGGAGTT TGATCCGGTC ATTTTCTCAA
601 TAGGACCCGT GGCGCTTCAC TGGTACGGCC GTCGACCCCC GGGGGATATC CCTGGGTGCA
661 GTACATCAGC ATGGGGCAAA TTCTTTCCAT CCCGATGATT GTCGCGGGTG TGATCATGAT
```

```
 721 GGTCTGGGCA TATCGTCGCA GCCCACAGCA ACACGTTTCC TGAGGAACCA TGAAACAGTA
 781 TTTAGAACTG ATGCAAAAAG TGCTCGACGA AGGCACACAG AAAAACGACC GTACCGGAAC
 841 CGGAACGCTT TCCATTTTTG GTCATCAGAT GCGTTTTAAC CTGCAAGATG GATTCCCGCT
 901 GGTGACAACT AAACGTTGCC ACCTGCGTTC CATCATCCAT GAACTGCTGT GGTTTCTGCA
 961 GGGCGACACT AACATTGCTT ATCTACACGA AAACAATGTC ACCATCTGGG ACGAATGGGC
1021 CGATGAAAAC GGCGACCTCG GGCCAGTGTA TGGTAAACAG TGGCGCGCCT GGCCAACGCC
1081 AGATGGTCGT CATATTGACC AGATCACTAC GGTACTGAAC CAGCTGAAAA ACGACCCGGA
1141 TTCGCGCCGC ATTATTGTTT CAGCGTGGAA CGTAGGCGAA CTGGATAAAA TGGCGCTGGC
1201 ACCGTGCCAT GCATTCCTCG AGCCC
```

SEQ 7 - BL21 *lgt* deletion sequence (highlighted in blue) inserted into pMT-ssB .

```
   1 CACCATAATG AAATAAGATC ACTACCGGGC GTATTTTTTG AGTTGTCGAG ATTTTCAGGA
  61 GCTAAGGAAG CTAAAATGGA GAAAAAAATC ACTGGATATA CCACCGTTGA TATATCCCAA
 121 TGGCATCGTA AAGAACATTT TGAGGCATTT CAGTCAGTTG CTCAATGTAC CTATAACCAG
 181 ACCGTTCAGC TGGATATTAC GGCCTTTTTA AAGACCGTAA AGAAAAATAA GCACAAGTTT
 241 TATCCGGCCT TTATTCACAT TCTTGCCCGC CTGATGAATG CTCATCCGGA ATTACGTATG
 301 GCAATGAAAG ACGGTGAGCT GGTGATATGG GATAGTGTTC ACCCTTGTTA CACCGTTTTC
 361 CATGAGCAAA CTGAAACGTT TTCATCGCTC TGGAGTGAAT ACCACGACGA TTTCCGGCAG
 421 TTTCTACACA TATATTCGCA AGATGTGGCG TGTTACGGTG AAAACCTGGC CTATTTCCCT
 481 AAAGGGTTTA TTGAGAATAT GTTTTTCGTC TCAGCCAATC CCTGGGTGAG TTTCACCAGT
 541 TTTGATTTAA ACGTGGCCAA TATGGACAAC TTCTTCGCCC CCGTTTTCAC CATGGGCAAA
 601 TATTATACGC AAGGCGACAA GGTGCTGATG CCGCTGGCGA TTCAGGTTCA TCATGCCGTT
 661 TGTGATGGCT TCCATGTCGG CAGAATGCTT AATGAATTAC AACAGTACTG CGATGAGTGG
 721 CAGGGCGGGG CGTAATTTTT TTAAGGCAGT TATTGGTGCC CATAAACGCC TGGTTGCTAC
 781 GCCTGAATAA GTGATAATAA GCGGATGAAT GGCAGAAATT CGATATTTTT TAGTTCTTTA
 841 GGCCCGTAGT CTGCAAATCC TTTTATGATT TTCTATCAAA CAAAAGAGGA AAATAGACCA
 901 GTTGCAATCC AAACGAGAGT CTAATAGAAT GAGGTCGAAA AGTAAATCGC GCGGGTTTGT
 961 TACTGATAAA GCAGGCAAGA CCTAAAATGT GTAAAGGGCA AAGTGTATAC TTTGGCGTCA
1021 CCCCTTACAT ATTTTAGGTC TTTTTTTATT GTGCGTAACT AACTTGCCAT CTTCAAACAG
1081 GAGGGCTGGA AGAAGCAGAC CGCTAACACA GTACATAAAA AAGGAGACAT GAACGATGAA
1141 CATCAAAAAG TTTGCAAAAC AAGCAACAGT ATTAACCTTT ACTACCGCAC TGCTGGCAGG
1201 AGGCGCAACT CAAGCGTTTG CGAAAGAAAC GAACCAAAAG CCATATAAGG AAACATACGG
1261 CATTTCCCAT ATTACACGCC ATGATATGCT GCAAATCCCT GAACAGCAAA AAAATGAAAA
1321 ATATCAAGTT CCTGAATTCG ATTCGTCCAC AATTAAAAAT ATCTCTTCTG CAAAAGGCCT
1381 GGACGTTTGG GACAGCTGGC CATTACAAAA CGCTGACGGC ACTGTCGCAA ACTATCACGG
1441 CTACCACATC GTCTTTGCAT TAGCCGGAGA TCCTAAAAAT GCGGATGACA CATCGATTTA
1501 CATGTTCTAT CAAAAAGTCG GCGAAACTTC TATTGACAGC TGGAAAAACG CTGGCCGCGT
1561 CTTTAAAGAC AGCGACAAAT TCGATGCAAA TGATTCTATC CTAAAAGACC AAACACAAGA
1621 ATGGTCAGGT TCAGCCACAT TTACATCTGA CGGAAAAATC CGTTTATTCT ACACTGATTT
1681 CTCCGGTAAA CATTACGGCA AACAAACACT GACAACTGCA CAAGTTAACG TATCAGCATC
1741 AGACAGCTCT TTGAACATCA ACGGTGTAGA GGATTATAAA TCAATCTTTG ACGGTGACGG
1801 AAAAACGTAT CAAAATGTAC AGCAGTTCAT CGATGAAGGC AACTACAGCT CAGGCGACAA
1861 CCATACGCTG AGAGATCCTC ACTACGTAGA AGATAAAGGC CACAAATACT TAGTATTTGA
1921 AGCAAACACT GGAACTGAAG ATGGCTACCA AGGCGAAGAA TCTTTATTTA ACAAAGCATA
1981 CTATGGCAAA AGCACATCAT TCTTCCGTCA AGAAAGTCAA AAACTTCTGC AAAGCGATAA
2041 AAAACGCACG GCTGAGTTAG CAAACGGCGC TCTCGGTATG ATTGAGCTAA ACGATGATTA
2101 CACACTGAAA AAAGTGATGA AACCGCTGAT TGCATCTAAC ACAGTAACAG ATGAAATTGA
2161 ACGCGCGAAC GTCTTTAAAA TGAACGGCAA ATGGTACCTG TTCACTGACT CCCGCGGATC
2221 AAAAATGACG ATTGACGGCA TTACGTCTAA CGATATTTAC ATGCTTGGTT ATGTTTCTAA
2281 TTCTTTAACT GGCCCATACA AGCCGCTGAA CAAAACTGGC CTTGTGTTAA AAATGGATCT
2341 TGATCCTAAC GATGTAACCT TTACTTACTC ACACTTCGCT GTACCTCAAG CGAAAGGAAA
2401 CAATGTCGTG ATTACAAGCT ATATGACAAA CAGAGGATTC TACGCAGACA AACAATCAAC
2461 GTTTGCGCCA AGCTTCCTGC TGAACATCAA AGGCAAGAAA ACATCTGTTG TCAAAGACAG
2521 CATCCTTGAA CAAGGACAAT TAACAGTTAA CAAATAAAAA CGCAAAAGAA AATGCCGATT
2581 GAGGCCAGTT TGCTCAGGCT CTCCCCGTGG AGGTAATAAT TGACGATATG ATCAGTAATA
2641 CGACTCACTA GTGGGCAGAT CTTCGAATGC ATCGCGCGCA CCGTACGTCT CGAGGAATGC
2701 ATGGCACGGT GCCAGCGCCA TTTTATCCAG TTCGCCTACG TTCCACGCTG AAACAATAAT
2761 GCGGCGCGAA TCCGGGTCGT TTTTCAGCTG GTTCAGTACC GTAGTGATCT GGTCAATATG
2821 ACGACCATCT GGCGTTGGCC AGGCGCGCCA CTGTTTACCA TACACTGGCC CGAGGTCGCC
2881 GTTTTCATCG GCCCATTCGT CCCAGATGGT GACATTGTTT TCGTGTAGAT AAGCAATGTT
2941 AGTGTCGCCC TGCAGAAACC ACAGCAGTTC ATGGATGATG GAACGCAGGT GGCAACGTTT
3001 AGTTGTCACC AGCGGGAATC CATCTTGCAG GTTAAAACGC ATCTGATGAC CAAAAATGGA
3061 AAGCGTTCCG GTTCCGGTAC GGTCGTTTTT CTGTGTGCCT TCGTCGAGCA CTTTTTGCAT
3121 CAGTTCTAAA TACTGTTTCA TGGTTCCTCA GGAAACGTGT TGCTGTGGGC TGCGACGATA
3181 TGCCCAGACC ATCATGATCA CACCCGCGAC AATCATCGGG ATGGAAAGAA TTTGCCCCAT
3241 GCTGATGTAC TGCACCCAGG GATATCCCCC GGGGGTCGAC GGCCGTACCA GTGAAGCGCC
3301 ACGGGTCCTA TTGAGAAAAT GACCGGATCA AACTCCGGAA AATGCAGATA GCTACTGGTC
3361 ATCTGTCACC ACAAGTTCTT GTTATTTCGC TGAAAGAGAA CAGCGATTGA AATGCGCGCC
```

```
3421 GCAGGTTTCA GGCGCTCCAA AGGTGCGAAT AATAGCACAA GGGGACCTGG CTGGTTGCCG
3481 GATACCGTTA AAAGATATGT ATATGATCCG CGCTATAACC CTCCGCGAAT CAGCCCGCCC
3541 ATGCCGCGAC GCTCCATAAA GGCTGCAACC TGATGGCGAA CTTCGGTCGC CAGTTGCGCT
3601 TCCAGACTAC GCTGCGCAAG ATTTTCTGCT TCGGCATAAT CAATGCGCCG CAGCAGGTAT
3661 TTTGCCCGCG CTACAGAACG TCCGTTCATA GACAGATGGC GATACCCAAG CCCAATGAGG
3721 ATTGCCACGC ACATGGGATC GCCCGCCATT TCACCGCACA AACGGAGATC GATTCCATGT
3781 ATTTCCGCTT CCCGGGCGAT CATCGCCAGA GCTCGTAACA TTGCAGGATG AAGACTGTCA
3841 TAAATGTTCG CCACCCGGGT ATTGTTGCGA TCAACGGCCA GAATGTATTG AGTCAGATCG
3901 TCTAGATACC TAGGTGAGCT CTGGTACCCT CTAGTCAAGG CCTGTCAGCC GTTAAGTGTT
3961 CCTGTGTCAC TGAAAATTGC TTTGAGAGGC TCTAAGGGCT TCTCAGTGCG TTACATCCCT
4021 GGCTTGTTGT CCACAACCGT TAAACCTTAA AAGCTTTAAA AGCCTTATAT ATTCTTTTTT
4081 TTCTTATAAA ACTTAAAACC TTAGAGGCTA TTTAAGTTGC TGATTTATAT TAATTTTATT
4141 GTTCAAACAT GAGAGCTTAG TACGTGAAAC ATGAGAGCTT AGTACGTTAG CCATGAGAGC
4201 TTAGGTTCGT TAAACATGAG AGCTTAGTAC GTTAAACATG AGAGCTTAGT ACGTGAAACA
4261 TGAGAGCTTA GTACGTACTA TCAACAGGTT GAACTGCTGA TCTTCAGATC TCCGCTTGCC
4321 CTCATCTGTT ACGCCGGCGG TAGCCGGCCA GCCTCGCAGA GCAGGATTCC CGTTGAGCAC
4381 CGCCAGGTGC GAATAAGGGA CAGTGAAGAA GGAACACCCG CTCGCGGGTG GGCCTACTTC
4441 ACCTATCCTG CCCGGCTGAC GCCGTTGGAT ACACCAAGGA AAGTCTACAC GAACCCTTTG
4501 GCAAAATCCT GTATATCGTG CGAAAAAGGA TGGATATACC GAAAAAATCG CTATAATGAC
4561 CCCGAAGCAG GGTTATGCAG CGGAAAAGCG CTGCTTCCCT GCTGTTTTGT GGAATATCTA
4621 CCGACTGGAA ACAGGCAAAT GCAGGAAATT ACTGAACTGA GGGGACAGGC GAGAGATCTG
4681 GCCTAGGCCG ACCGAATAAA TACCTGTGAC GGAAGATCAC TTCGCAGAAT AAATAAATCC
4741 TGGTGTCCCT GTTGATACCG GGAAGCCCTG GGCCAACTTT TGGCGAAAAT GAGACGTTGA
4801 TCGGCACGTA AGAGGTTCCA ACTTT
```

SEQ 8 - *lgt* deletion with insertion of *loxP* sites and Tn5 kanamycin resistance gene (highlighted in blue). The coding region for the kanamycin resistance gene is underlined

```
   1 TCTAGACGAT CTGACTCAAT ACATTCTGGC CGTTGATCGC AACAATACCC GGGTGGCGAA
  61 CATTTATGAC AGTCTTCATC CTGCAATGTT ACGAGCTCTG GCGATGATCG CCCGGGAAGC
 121 GGAAATACAT GGAATCGATC TCCGTTTGTG CGGTGAAATG GCGGGCGATC CCATGTGCGT
 181 GGCAATCCTC ATTGGGCTTG GGTATCGCCA TCTGTCTATG AACGGACGTT CTGTAGCGCG
 241 GGCAAAATAC CTGCTGCGGC GCATTGATTA TGCCGAAGCA GAAAATCTTG CGCAGCGTAG
 301 TCTGGAAGCG CAACTGGCGA CCGAAGTTCG CCATCAGGTT GCAGCCTTTA TGGAGCGTCG
 361 CGGCATGGGC GGGCTGATTC GCGGAGGGTT ATAGCGCGGA TCATATACAT ATCTTTTAAC
 421 GGTATCCGGC AACCAGCCAG GTCCCCTTGT GCTATTATTC GCACCTTTGG AGCGCCTGAA
 481 ACCTGCGGCG CGCATTTCAA TCGCTGTTCT CTTTCAGCGA AATAACAAGA ACTTGTGGTG
 541 ACAGATGACC AGTAGCTATC TGCATTTTCC GGAGTTTGAT CCGGTCATTT TCTCAATAGG
 601 ACCCGTGGCG CTTCACTGGT ACGGCCGTCG ACTATAACTT CGTATAGCAT ACATTATACG
 661 AAGTTATTGC CACGGGCGGC CGGGAAAGCC ACGTTGTGTC TCAAAATCTC TGATGTTACA
 721 TTGCACAAGA TAAAAATATA TCATCATGAA CAATAAAACT GTCTGCTTAC ATAAACAGTA
 781 ATACAAGGGG TGTTATGAGC CATATTCAAC GGGAAACGTC TTGCTCGAGG CCGCGATTAA
 841 ATTCCAACAT GGATGCTGAT TTATATGGGT ATAAATGGGC TCGCGATAAT GTCGGGCAAT
 901 CAGGTGCGAC AATCTATCGA TTGTATGGGA AGCCCGATGC GCCAGAGTTG TTTCTGAAAC
 961 ATGGCAAAGG TAGCGTTGCC AATGATGTTA CAGATGAGAT GGTCAGACTA AACTGGCTGA
1021 CGGAATTTAT GCCTCTTCCG ACCATCAAGC ATTTTATCCG TACTCCTGAT GATGCATGGT
1081 TACTCACCAC TGCGATCCCC GGGAAAACAG CATTCCAGGT ATTAGAAGAA TATCCTGATT
1141 CAGGTGAAAA TATTGTTGAT GCGCTGGCAG TGTTCCTGCG CCGGTTGCAT TCGATTCCTG
1201 TTTGTAATTG TCCTTTTAAC AGCGATCGCG TATTTCGTCT CGCTCAGGCG CAATCACGAA
1261 TGAATAACGG TTTGGTTGAT GCGAGTGATT TTGATGACGA GCGTAATGGC TGGCCTGTTG
1321 AACAAGTCTG GAAAGAAATG CATAAGCTCT TGCCATTCTC ACCGGATTCA GTCGTCACTC
1381 ATGGTGATTT CTCACTTGAT AACCTTATTT TTGACGAGGG GAAATTAATA GGTTGTATTG
1441 ATGTTGGACG AGTCGGAATC GCAGACCGAT ACCAGGATCT TGCCATCCTA TGGAACTGCC
1501 TCGGTGAGTT TTCTCCTTCA TTACAGAAAC GGCTTTTTCA AAAATATGGT ATTGATAATC
1561 CTGATATGAA TAAATTGCAG TTTCATTTGA TGCTCGATGA GTTTTTCTAA TCAGAATTGG
1621 TTAATTGGTT GTAACACTGG CAGAGCATTA CGCTGACTTG ACGGGACGGC GGCTTTGTTG
1681 AATAAATCGA ACTTTTGCTG AGTTGAAGGA TCAGATCACG CATCTTCCCG ACAACGCAGA
1741 CCGTTCCGTG GCAAAGCAAA AGTTCAAAAT CACCAACTGG TCCACCTACA ACAAAGCTCT
1801 CATCAACCGT GGCTCCCTCA CTTTCTGGCT GGATGATGGG GCGATTCAGG CCTGGTATGA
1861 GTCAGCAACA CCTTCTTCAC GAGGCAGACC TCAGCGCCCC CGGCCGCATA ACTTCGTATA
1921 GCATACATTA TACGAAGTTA TTGATATCCC TGGGTGCAGT ACATCAGCAT GGGGCAAATT
1981 CTTTCCATCC CGATGATTGT CGCGGGTGTG ATCATGATGG TCTGGGCATA TCGTCGCAGC
2041 CCACAGCAAC ACGTTTCCTG AGGAACCATG AAACAGTATT TAGAACTGAT GCAAAAAGTG
2101 CTCGACGAAG GCACACAGAA AAACGACCGT ACCGGAACCG GAACGCTTTC CATTTTTGGT
2161 CATCAGATGC GTTTTAACCT GCAAGATGGA TTCCCGCTGG TGACAACTAA ACGTTGCCAC
2221 CTGCGTTCCA TCATCCATGA ACTGCTGTGG TTTCTGCAGG GCGACACTAA CATTGCTTAT
2281 CTACACGAAA ACAATGTCAC CATCTGGGAC GAATGGGCCG ATGAAAACGG CGACCTCGGG
2341 CCAGTGTATG GTAAACAGTG GCGCGCCTGG CCAACGCCAG ATGGTCGTCA TATTGACCAG
2401 ATCACTACGG TACTGAACCA GCTGAAAAAC GACCCGGATT CGCGCCGCAT TATTGTTTCA
2461 GCGTGGAACG TAGGCGAACT GGATAAAATG GCGCTGGCAC CGTGCCATGC ATTCCTCGAG
```

SEQ 9 - *lgt* deletion in BL21 leaving only the *loxP* site (highlighted in blue) following removal of the kanamycin resistance gene by Cre-mediated recombination. The sequence was obtained from chromosomal DNA of *E. coli* strain MMS1742.

```
   1 CGATCTGACT CAATACATTC TGGCCGTTGA TCGCAACAAT ACCCGGGTGG CGAACATTTA
  61 TGACAGTCTT CATCCTGCAA TGTTACGAGC TCTGGCGATG ATCGCCCGGG AAGCGGAAAT
 121 ACATGGAATC GATCTCCGTT TGTGCGGTGA AATGGCGGGC GATCCCATGT GCGTGGCAAT
 181 CCTCATTGGG CTTGGGTATC GCCATCTGTC TATGAACGGA CGTTCTGTAG CGCGGGCAAA
 241 ATACCTGCTG CGGCGCATTG ATTATGCCGA AGCAGAAAAT CTTGCGCAGC GTAGTCTGGA
 301 AGCGCAACTG GCGACCGAAG TTCGCCATCA GGTTGCAGCC TTTATGGAGC GTCGCGGCAT
 361 GGGCGGGCTG ATTCGCGGAG GGTTATAGCG CGGATCATAT ACATATCTTT TAACGGTATC
 421 CGGCAACCAG CCAGGTCCCC TTGTGCTATT ATTCGCACCT TTGGAGCGCC TGAAACCTGC
 481 GGCGCGCATT TCAATCGCTG TTCTCTTTCA GCGAAATAAC AAGAACTTGT GGTGACAGAT
 541 GACCAGTAGC TATCTGCATT TTCCGGAGTT TGATCCGGTC ATTTTCTCAA TAGGACCCGT
 601 GGCGCTTCAC TGGTACGGCC GTCGACTATA ACTTCGTATA GCATACATTA TACGAAGTTA
 661 TTGATATCCC TGGGTGCAGT ACATCAGCAT GGGGCAAATT CTTTCCATCC CGATGATTGT
 721 CGCGGGTGTG ATCATGATGG TCTGGGCATA TCGTCGCAGC CCACAGCAAC ACGTTTCCTG
 781 AGGAACCATG AAACAGTATT TAGAACTGAT GCAAAAAGTG CTCGACGAAG GCACACAGAA
 841 AAACGACCGT ACCGGAACCG GAACGCTTTC CATTTTTGGT CATCAGATGC GTTTTAACCT
 901 GCAAGATGGA TTCCCGCTGG TGACAACTAA ACGTTGCCAC CTGCGTTCCA TCATCCATGA
 961 ACTGCTGTGG TTTCTGCAGG GCGACACTAA CATTGCTTAT CTACACGAAA ACAATGTCAC
1021 CATCTGGGAC GAATGGGCCG ATGAAAACGG CGACCTCGGG CCAGTGTATG GTAAACAGTG
1081 GCGCGCCTGG CCAACGCCAG ATGGTCGTCA TATTGACCAG ATCACTACGG TACTGAACCA
1141 GCTGAAAAAC GACCCGGATT CGCGCCGCAT TATTGTTTCA GCGTGGAACG TAGGCGAACT
1201 GGATAAAATG GCGCTGGCAC CGTGCCATGC ATTC
```

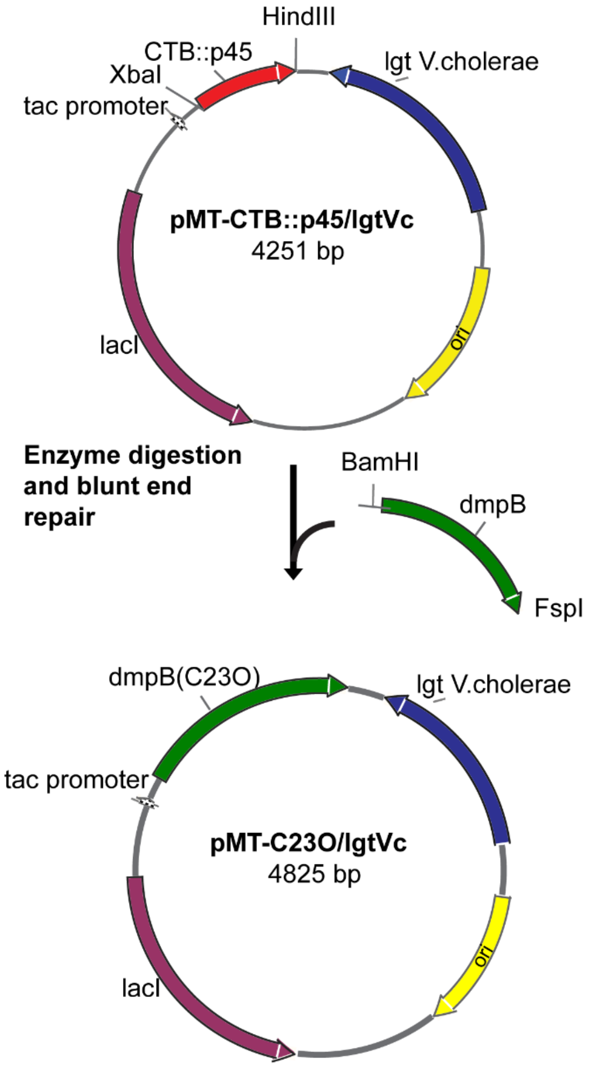

Figure S1. Cloning strategy of pMT-C23O/lgtVc plasmid used in the stability test in E. coli strain MMS1742. The *ctxB* fusion gene in the plasmid pMT-CTB::p45/lgtVc was substituted with the *dmpB* gene encoding a catechol 2,3 dioxygenase (C23O). The plasmid was digested and blunt end repaired as shown prior to insertion of the dmpB gene. Positive clones were selected on the basis of IPTG-inducible C23O expression detected by the production of 2-hydroxymuconic semialdehyde from catechol.

SUPPLEMENTARY DATA SECTION 2

Sequence data from the construction of the *V. cholerae* strain JS1569 *lgt*-deleted derivative MMS1663

SEQ 10- *lgt* gene amplified from *Escherichia coli* using primers lgt ECf and lgt ECr (underlined). The coding region is highlighted in blue. The PCR product was digested with EcoRI and HindIII, blunt-end repaired and inserted into temperature-sensitive vector derived from pSC101 (see cloning strategy in **Figure S2**):

```
   1 GGGGGGTCTC GAATTCGCTG CGGCGCATTG ATTATGCCGA AGCAGAAAAT CTTGCGCAGC
  61 GTAGTCTGGA AGCGCAACTG GCGACCGAAG TTCGCCATCA GGTTGCAGCC TTTATGGAGC
 121 GTCGCGGCAT GGGCGGGCTG ATTCGCGGAG GGTTATAGCG CGGATCATAT ACATATCTTT
 181 TAACGGTATC CGGCAACCAG CCAGGTCCCC TTGTGCTATT ATTCGCACCT TTGGAGCGCC
 241 TGAAACCTGC GGCGCGCATT TCAATCGCTG TTCTCTTTCA GCGAAATAAC AAGAACTTGT
 301 GGTGACAGAT GACCAGTAGC TATCTGCATT TTCCGGAGTT TGATCCGGTC ATTTTCTCAA
 361 TAGGACCCGT GGCGCTTCAC TGGTACGGCC TGATGTATCT GGTGGGTTTC ATTTTTGCAA
 421 TGTGGCTGGC AACACGACGG GCGAATCGTC CGGGCAGCGG CTGGACCAAA AATGAAGTTG
 481 AAAACTTACT CTATGCGGGC TTCCTCGGCG TCTTCCTCGG GGGACGTATT GGTTATGTTC
 541 TGTTCTACAA TTTCCCGCAG TTTATGGCCG ATCCGCTGTA TCTGTTCCGT GTCTGGGACG
 601 GCGGCATGTC TTTCCACGGC GGCCTGATTG GCGTTATCGT GGTGATGATT ATCTTCGCCC
 661 GCCGTACTAA ACGTTCCTTC TTCCAGGTCT CTGATTTTAT CGCACCACTC ATTCCGTTTG
 721 GTCTTGGTGC CGGGCGTCTG GGCAACTTTA TTAACGGTGA ATTGTGGGGC CGCGTTGACC
 781 CGAACTTCCC GTTTGCCATG CTGTTCCCTG GCTCCCGTAC AGAAGATATT TTGCTGCTGC
 841 AAACCAACCC GCAGTGGCAA TCCATTTTCG ACACTTACGG TGTGCTGCCG CGCCACCCAT
 901 CACAGCTTTA CGAGCTGCTG CTGGAAGGTG TGGTGCTGTT TATTATCCTC AACCTGTATA
 961 TTCGTAAACC ACGCCCAATG GGAGCTGTCT CAGGTTTGTT CCTGATTGGT TACGGCGCGT
1021 TTCGCATCAT TGTTGAGTTT TTCCGCCAGC CCGACGCGCA GTTTACCGGT GCCTGGGTGC
1081 AGTACATCAG CATGGGGCAA ATTCTTTCCA TCCCGATGAT TGTCGCGGGT GTGATCATGA
1141 TGGTCTGGGC ATATCGTCGC AGCCCACAGC AACACGTTTC CTGAGGAACC ATGAAACAGT
1201 ATTTAGAACT GATGCAAAAA GTGCTCGACG AAGGCACACA GAAGCTTGAG ACCCCCC
```

SEQ 11- Region of *V. cholerae* chromosome encoding *lgt* (coding region underlined) with the deleted region highlighted in blue:

```
   1 GCGGTACCGG CATTGGGGTA ATCTCAAGTT TGGCCGGTAT TGGCGGCGGT TCTTTATCGG
  61 TGCCATTTTT GAATCGACAT GGCATTGAAA TGAAAAAGGC GATAGGTTCT TCATCGGTCT
 121 GTGGTTTTGC GATTGCGATA TCGGGCATGA TTGGTTTTAT TTTGCACGGT TATCAAGTGG
 181 AGAACTTGCC ACAATACAGC CTTGGTTATG TTTATTTACC TGCATTGTTA GCGATTGCTA
 241 CAACATCGAT GCTTACCACG CGAATTGGCG CTAAACTTGC CACCCAAATG CCAACAGCAA
 301 GGCTTAAGCG ATTCTTTGCC ATTTTTTTAA TGTGCGTCGC TGTGACCATG TTGTTCCAGT
 361 AATACTCATT GTTTATAGAG AAGGTTTGTT ATGCCTCAGG GTTATCTGCA GTTTCCCAAT
 421 ATTGACCCCG TATTGTTTTC GATCGGCCCT CTAGCGGTGC GCTGGTATGG CTTGATGTAT
 481 TTGGTGGGTT TCCTTTTTGC TATGTGGTTG GCCAATCGCC GAGCGGATCG CGCGGGCAGT
 541 GGTTGGACGC GTGAGCAAGT CTCTGACTTG TTATTCGCCG GCTTTTTAGG TGTAGTGATC
 601 GGTGGCCGAG TTGGTTATGT GATCTTCTAC AATTTTGATC TGTTCCTTGC TGACCCTCTT
```

661 TATTTATTCA AAGTGTGGAC TGGCGGCATG TCCTTCCACG GCGGCTTATT GGGTGTGATC
721 ACCGCCATGT TCTGGTATGC GCGTAAAAAC CAACGCACCT TCTTTGGTGT GGCCGATTTT
781 GTTGCCCCTT TAGTGCCATT CGGTTTGGGG ATGGGACGTA TCGGTAACTT TATGAATAGT
841 GAACTTTGGG GACGAGTAAC GGATGTGCCT TGGGCTTTTG TATTCCCTAA TGGTGGCCCA
901 CTGCCGCGCC ATCCTTCACA GCTTTATGAA TTCGCCTTAG AAGGCGTGGT GCGTATCCGG
961 TCTGTTCTTT ATTCTTAATT GGTTTATTGG TAAACCTCGT CCGCTAGGCA ACTGTTTTTA
1021 GCTGGATACG GTACATTCCG CTTCCTTGTG GAATACGTCC GTGAGCCAGA TGCTCAGTTG
1081 GGTCTGTTTG GTGGCTTCAT TTCAATGGGG CAAATCCTCT CCTTACCTAT GGTGATCATC
1141 GGTATTTTGA TGATGGTTTG GTCTTACAAG CGCGGTTTGT ATCAAGACCG TGTAGCAGCA
1201 AAATAGGGTA GTTAGGTGAG ACAGTATTTA GATCTTTGTC AGCGCATCGT CGATCAAGGT
1261 GTTTGGGTTG AAAATGAACG AACGGGCAAG CGTTGTTTGA CTGTGATTAA TGCCGATTTG
1321 ACCTACGATG TGGGCAACAA TCAGTTTCCT CTAGTGACTA CACGCAAGAG TTTTTGGAAA
1381 GCTGCCGTAG CCGAGTTGCT CGGCTATATT CGTGGTTACG ATAATGCGGC GGATTTTCGC
1441 CAATTAGGTA CCAAAACCTG GGATGCTAAT GCCAATTTAA ACCAAGCATG GCTCAACAAT
1501 CCTTACCGTA AAGGTGAGGA TGACATGGGA CGCGTGTATG GTGTTCAGGG TAGAGCTTGG
1561 GCTAAGCCTG ATGGTGGTCA TATTGACCAG TTGAAAAAGA TTGTTGATGA TTTGAGCCGT
1621 GGCGTTGATG ACCGAGGTGA AATTCTTAAC TTCTACAATC CGGGTGAATT TCACATGGGG
1681 TGTTTGCGCC CTTGCATGTA CAGCCATCAT TTTTCATTGC TGGGGGATAC CTTGTATCTC
1741 AACAGTACTC AGCGTTCATG TGATGTGCCC TTGGGGTTGA ATTTCAACAT GGTGCAGGTT
1801 TATGTGTTCC TTGCGCTGAT GG

SEQ12 - Amplified DNA using primer pair VCD1/ VCD2 (Primer sequences underlined):

1 GCGATTGCGA TATCGGGCAT GATTGGTTTT ATTTTGCACG GTTATCAAGT GGAGAACTTG
51 CCACAATACA GCCTTGGTTA TGTTTATTTA CCTGCATTGT TAGCGATTGC TACAACATCG
101 ATGCTTACCA CGCGAATTGG CGCTAAACTT GCCACCCAAA TGCCAACAGC AAGGCTTAAG
151 CGATTCTTTG CCATTTTTTT AATGTGCGTC GCTGTGACCA TGTTGTTCCA GTAATACTCA
201 TTGTTTATAG AGAAGGTTTG TTATGCCTCA GGGTTATCTG CAGTTTCCCA ATATTGACCC
251 CGTATTGTTT TCGATCGGCC CTCTAGCGGT GCGC

SEQ 13 - Amplified DNA using primer pair VCD3/ VCD4 (primer sequences underlined)

The PvuII site replacing the *lgt* gene is highlighted:

1 CGATCGGCCC TCTAGCGGTG CGCAGCTGTC GCGGTTTGTA TCAAGACCGT GTAGCAGCAA
61 AATAGGGTAG TTAGGTGAGA CAGTATTTAG ATCTTTGTCA GCGCATCGTC GATCAAGGTG
121 TTTGGGTTGA AAATGAACGA ACGGGCAAGC GTTGTTTGAC TGTGATTAAT GCCGATTTGA
181 CCTACGATGT GGGCAACAAT CAGTTTCCTC TAGTGACTAC ACGCAAGAGT TTTTGGAAAG
241 CTGCCGTAGC CGAGTTGCTC GGCTATATTC GTGGTTACGA TAATGCGGCG GATTTTCGCC
301 AATTAGGTAC CAAAACCTGG GATGCTAATG CCAATTTAAA CCAAGCATGG CTCAACAATC
361 CTTACCGTAA AGGTGAGGAT GACATGGGAC GCGTGTATGG TGTTCAGGGT AGAGCTTGGG
421 CTAAGCCTGA TGGTGGTCAT ATTGACCAGT TGAAAAAGAT TGTTGATGAT TTGAGCCGTG
481 GCGTTGATGA CCGAGGTGAA ATTCTTAACT TCTACAATCC GGGTGAATTT CACATGGGGT
541 GTTTGCGCCC TTGCATGTAC AGCCGGATCC CCC

SEQ 14 - Amplified DNA following primerless PCR and amplification with VCD1 and VCD4. A unique PvuII (highlighted in blue) site replaces the *lgt* gene sequence:

1 GCGATTGCGA TATCGGGCAT GATTGGTTTT ATTTTGCACG GTTATCAAGT GGAGAACTTG

```
 61 CCACAATACA GCCTTGGTTA TGTTTATTTA CCTGCATTGT TAGCGATTGC TACAACATCG
121 ATGCTTACCA CGCGAATTGG CGCTAAACTT GCCACCCAAA TGCCAACAGC AAGGCTTAAG
181 CGATTCTTTG CCATTTTTTT AATGTGCGTC GCTGTGACCA TGTTGTTCCA GTAATACTCA
241 TTGTTTATAG AGAAGGTTTG TTATGCCTCA GGGTTATCTG CAGTTTCCCA ATATTGACCC
301 CGTATTGTTT TCGATCGGCC CTCTAGCGGT GCGCAGCTG  CGCGGTTTGT ATCAAGACCG
361 TGTAGCAGCA AAATAGGGTA GTTAGGTGAG ACAGTATTTA GATCTTTGTC AGCGCATCGT
421 CGATCAAGGT GTTTGGGTTG AAAATGAACG AACGGGCAAG CGTTGTTTGA CTGTGATTAA
481 TGCCGATTTG ACCTACGATG TGGGCAACAA TCAGTTTCCT CTAGTGACTA CACGCAAGAG
541 TTTTTGGAAA GCTGCCGTAG CCGAGTTGCT CGGCTATATT CGTGGTTACG ATAATGCGGC
601 GGATTTTCGC CAATTAGGTA CCAAAACCTG GGATGCTAAT GCCAATTTAA ACCAAGCATG
661 GCTCAACAAT CCTTACCGTA AAGGTGAGGA TGACATGGGA CGCGTGTATG GTGTTCAGGG
721 TAGAGCTTGG GCTAAGCCTG ATGGTGGTCA TATTGACCAG TTGAAAAAGA TTGTTGATGA
781 TTTGAGCCGT GGCGTTGATG ACCGAGGTGA AATTCTTAAC TTCTACAATC CGGGTGAATT
841 TCACATGGGG TGTTTGCGCC CTTGCATGTA CAGCCGGATC CCCC
```

The fragment was inserted into the pML-ssB suicide vector using EcoRV and BamHI. The EcoRV/SalI fragment carrying Kanamycin gene the flanked by loxP sites was blunt-end repaired and inserted into the PvuII site.

SEQ 15- DNA fragment carrying the kanamycin resistance cassette and *loxP* sites (highlighted) The kanamycin resistance gene is underlined:

```
   1 GCGATTGCGA TATCGGGCAT GATTGGTTTT ATTTTGCACG GTTATCAAGT GGAGAACTTG
  61 CCACAATACA GCCTTGGTTA TGTTTATTTA CCTGCATTGT TAGCGATTGC TACAACATCG
 121 ATGCTTACCA CGCGAATTGG CGCTAAACTT GCCACCCAAA TGCCAACAGC AAGGCTTAAG
 181 CGATTCTTTG CCATTTTTTT AATGTGCGTC GCTGTGACCA TGTTGTTCCA GTAATACTCA
 241 TTGTTTATAG AGAAGGTTTG TTATGCCTCA GGGTTATCTG CAGTTTCCCA ATATTGACCC
 301 CGTATTGTTT TCGATCGGCC CTCTAGCGGT GCGCAGATCA ATAACTTCGT ATAATGTATG
 361 CTATACGAAG TTATGCGGCC GGGGGCGCTG AGGTCTGCCT CGTGAAGAAG GTGTTGCTGA
 421 CTCATACCAG GCCTGAATCG CCCCATCATC CAGCCAGAAA GTGAGGGAGC CACGGTTGAT
 481 GAGAGCTTTG TTGTAGGTGG ACCAGTTGGT GATTTTGAAC TTTTGCTTTG CCACGGAACG
 541 GTCTGCGTTG TCGGGAAGAT GCGTGATCTG ATCCTTCAAC TCAGCAAAAG TTCGATTTAT
 601 TCAACAAAGC CGCCGTCCCG TCAAGTCAGC GTAATGCTCT GCCAGTGTTA CAACCAATTA
 661 ACCAATTCTG ATTAGAAAAA CTCATCGAGC ATCAAATGAA ACTGCAATTT ATTCATATCA
 721 GGATTATCAA TACCATATTT TTGAAAAAGC CGTTTCTGTA ATGAAGGAGA AAACTCACCG
 781 AGGCAGTTCC ATAGGATGGC AAGATCCTGG TATCGGTCTG CGATTCCGAC TCGTCCAACA
 841 TCAATACAAC CTATTAATTT CCCCTCGTCA AAAATAAGGT TATCAAGTGA GAAATCACCA
 901 TGAGTGACGA CTGAATCCGG TGAGAATGGC AAGAGCTTAT GCATTTCTTT CCAGACTTGT
 961 TCAACAGGCC AGCCATTACG CTCGTCATCA AAATCACTCG CATCAACCAA ACCGTTATTC
1021 ATTCGTGATT GCGCCTGAGC GAGACGAAAT ACGCGATCGC TGTTAAAAGG ACAATTACAA
1081 ACAGGAATCG AATGCAACCG GCGCAGGAAC ACTGCCAGCG CATCAACAAT ATTTTCACCT
1141 GAATCAGGAT ATTCTTCTAA TACCTGGAAT GCTGTTTTCC CGGGGATCGC AGTGGTGAGT
1201 AACCATGCAT CATCAGGAGT ACGGATAAAA TGCTTGATGG TCGGAAGAGG CATAAATTCC
1261 GTCAGCCAGT TTAGTCTGAC CATCTCATCT GTAACATCAT TGGCAACGCT ACCTTTGCCA
1321 TGTTTCAGAA ACAACTCTGG CGCATCGGGC TTCCCATACA ATCGATAGAT TGTCGCACCT
1381 GATTGCCCGA CATTATCGCG AGCCCATTTA TACCCATATA AATCAGCATC CATGTTGGAA
1441 TTTAATCGCG GCCTCGAGCA AGACGTTTCC CGTTGAATAT GGCTCATAAC ACCCCTTGTA
1501 TTACTGTTTA TGTAAGCAGA CAGTTTTATT GTTCATGATG ATATATTTTT ATCTTGTGCA
1561 ATGTAACATC AGAGATTTTG AGACACAACG TGGCTTTCCC GGCCGCCCGT GGCAATAACT
1621 TCGTATAATG TATGCTATAC GAAGTTATAG TCGACTGTCG CGGTTTGTAT CAAGACCGTG
1681 TAGCAGCAAA ATAGGGTAGT TAGGTGAGAC AGTATTTAGA TCTTTGTCAG CGCATCGTCG
```

```
1741 ATCAAGGTGT TTGGGTTGAA AATGAACGAA CGGGCAAGCG TTGTTTGACT GTGATTAATG
1801 CCGATTTGAC CTACGATGTG GGCAACAATC AGTTTCCTCT AGTGACTACA CGCAAGAGTT
1861 TTTGGAAAGC TGCCGTAGCC GAGTTGCTCG GCTATATTCG TGGTTACGAT AATGCGGCGG
1921 ATTTTCGCCA ATTAGGTACC AAAACCTGGG ATGCTAATGC CAATTTAAAC CAAGCATGGC
1981 TCAACAATCC TTACCGTAAA GGTGAGGATG ACATGGGACG CGTGTATGGT GTTCAGGGTA
2041 GAGCTTGGGC TAAGCCTGAT GGTGGTCATA TTGACCAGTT GAAAAAGATT GTTGATGATT
2101 TGAGCCGTGG CGTTGATGAC CGAGGTGAAA TTCTTAACTT CTACAATCCG GGTGAATTTC
2161 ACATGGGGTG TTTGCGCCCT TGCATGTACA GCCGGATCCC CC
```

SEQ 16 - Chromosomal fragment from MMS1663 in which the kanamycin resistance cassette has been removed by Cre mediated recombination leaving a single *loxP* site (highlighted):

```
   1 GCGGTACCGG CATTGGGGTA ATCTCAAGTT TGGCCGGTAT TGGCGGCGGT TCTTTATCGG
  61 TGCCATTTTT GAATCGACAT GGCATTGAAA TGAAAAAGGC GATAGGTTCT TCATCGGTCT
 121 GTGGTTTTGC GATTGCGATA TCGGGCATGA TTGGTTTTAT TTTGCACGGT TATCAAGTGG
 181 AGAACTTGCC ACAATACAGC CTTGGTTATG TTTATTTACC TGCATTGTTA GCGATTGCTA
 241 CAACATCGAT GCTTACCACG CGAATTGGCG CTAAACTTGC CACCCAAATG CCAACAGCAA
 301 GGCTTAAGCG ATTCTTTGCC ATTTTTTTAA TGTGCGTCGC TGTGACCATG TTGTTCCAGT
 361 AATACTCATT GTTTATAGAG AAGGTTTGTT ATGCCTCAGG GTTATCTGCA GTTTCCCAAT
 421 ATTGACCCCG TATTGTTTTC GATCGGCCCT CTAGCGGTGC GCAGATCAAT AACTTCGTAT
 481 AATGTATGCT ATACGAAGTT ATAGTCGACT GTCGCGGTTT GTATCAAGAC CGTGTAGCAG
 541 CAAAATAGGG TAGTTAGGTG AGACAGTATT TAGATCTTTG TCAGCGCATC GTCGATCAAG
 601 GTGTTTGGGT TGAAAATGAA CGAACGGGCA AGCGTTGTTT GACTGTGATT AATGCCGATT
 661 TGACCTACGA TGTGGGCAAC AATCAGTTTC CTCTAGTGAC TACACGCAAG AGTTTTTGGA
 721 AAGCTGCCGT AGCCGAGTTG CTCGGCTATA TTCGTGGTTA CGATAATGCG GCGGATTTTC
 781 GCCAATTAGG TACCAAAACC TGGGATGCTA ATGCCAATTT AAACCAAGCA TGGCTCAACA
 841 ATCCTTACCG TAAAGGTGAG GATGACATGG GACGCGTGTA TGGTGTTCAG GGTAGAGCTT
 901 GGGCTAAGCC TGATGGTGGT CATATTGACC AGTTGAAAAA GATTGTTGAT GATTTGAGCC
 961 GTGGCGTTGA TGACCGAGGT GAAATTCTTA ACTTCTACAA TCCGGGTGAA TTTCACATGG
1021 GGTGTTTGCG CCCTTGCATG TACAGCCATC ATTTTTCATT GCTGGGGGAT ACCTTGTATC
1081 TCAACAGTAC TCAGCGTTCA TGTGATGTGC CCTTGGGGTT GAATTTCAAC ATGGTGCAGG
1141 TTTATGTGTT CCTTGCGCTG ATGG
```

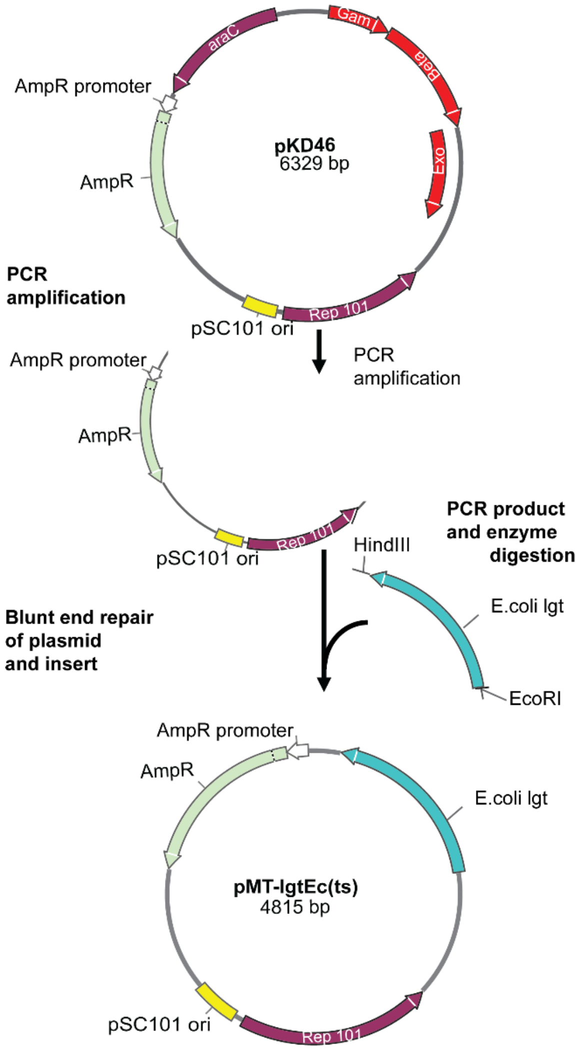

Figure S2. **Construction of the maintenance plasmid pMT- lgtEc(ts) plasmid.** The construction of the maintenance plasmid for *lgt*-deleted derivatives of *V. cholerae* was done in the same way as for the reciprocal plasmid pMT-lgtVc(ts). pKD46 is derived from the temperature sensitive pSC101. The plasmid was amplified to obtain the replicon and ligated together with the *lgt*-encoding region amplified from *E. coli* with PCR primers lgt ECf and lgt ECr.

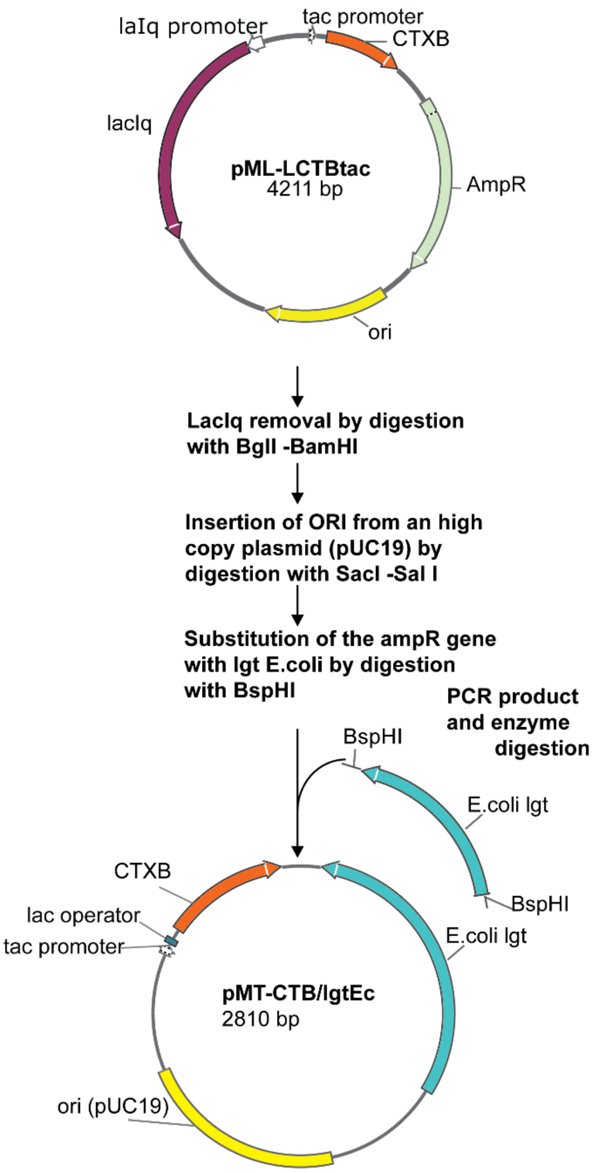

Figure S3. Summary of the construction of the CTB production plasmid pMT-CTB/lgtEc. The pML-LCTBtac expression plasmid carrying *ctxB* and derived from pAFtac1 was used as a starting plasmid. In order to obtain constitutive expression the lacIq gene was removed by digestion with BamHI and BglII and relegation. To raise the copy number of the plasmid the origin of replication from pBR322 was replaced by that from pUC19 and finally, the ampicillin resistance gene was replaced by the lgt gene derived from *E. coli*.
